# Exploring neuroendocrine influences on the sensorimotor-association axis in a female and a male individual

**DOI:** 10.1162/imag_a_00474

**Published:** 2025-02-18

**Authors:** Bianca Serio, Deniz Yilmaz, Laura Pritschet, Hannah Grotzinger, Emily G. Jacobs, Simon B. Eickhoff, Sofie L. Valk

**Affiliations:** Institute of Neuroscience and Medicine, Brain & Behavior (INM-7), Research Centre Jülich, Jülich, Germany; Max Planck Institute for Human Cognitive and Brain Sciences, Leipzig, Germany; Max Planck School of Cognition, Leipzig, Germany; Institute of Systems Neuroscience, Medical Faculty, Heinrich Heine University Düsseldorf, Düsseldorf, Germany; Palo Alto High School, Palo Alto, CA, United States; Department of Psychological and Brain Sciences, University of California, Santa Barbara, CA, United States

**Keywords:** brain organization, functional connectivity, steroid hormones, dense sampling, individual variability

## Abstract

Human neuroimaging studies consistently show multimodal patterns of variabilityalong a key principle of macroscale cortical organization—thesensorimotor-association (S-A) axis. However, little is known about day-to-dayfluctuations in functional activity along this axis within an individual,including sex-specific neuroendocrine factors contributing to such transientchanges. We leveraged data from two densely sampled healthy young adults, onefemale and one male, to investigate intra-individual daily variability along theS-A axis, which we computed as our measure of functional cortical organizationby reducing the dimensionality of functional connectivity matrices. Dailyvariability was greatest in temporal limbic and ventral prefrontal regions inboth participants, and was more strongly pronounced in the male subject. Next,we probed local- and system-level effects of steroid hormones and self-reportedperceived stress on functional organization. Beyond shared patterns of effects,our findings revealed subtle and unique associations between neuroendocrinefluctuations and intra-individual variability along the S-A axis in the femaleand male participants. In sum, our study points to neuroendocrine factors aspossible modulators of intra-individual variability in functional brainorganization, highlighting the need for further research in larger samples toassess the sex specificity of these effects.

## 1 Introduction

Patterns of functional connectivity are considered to be broadly stable, trait-likefeatures of the human brain, both within and between individuals (Damoiseaux et al., 2006;Gratton et al., 2018;Power et al., 2011). Inparticular, ubiquitous patterns of functional connectivity across cortical structureand function seem to reflect a major principle of brain organization, also known asthe sensorimotor-association (S-A) axis (Margulies et al., 2016;Sydnor et al., 2021). More specifically, this axis offunctional organization differentiates unimodal primary regions, such as the visualand the sensorimotor cortices, from heteromodal association regions involved inhigher order cognitive functions, such as regions in frontal, parietal, and temporalcortices, including the medial prefrontal cortex, superior temporal sulcus, andprecuneus. However, beyond the consistency and robustness of functional networksarranged along this axis lies a subtle—yet notable—degree ofintra-individual variability (Park etal., 2012), suggesting that the S-A axis also has dynamic properties,even at rest. Given that the brain is an endocrine organ, susceptible to transientendogenous fluctuations in the levels of different steroid hormones across sexes(Banks, 2012), suchfluctuations may influence the dynamic reconfiguration of functional networksunderpinning intra-individual variability, ultimately supporting flexible cognitionand behavior (Shine & Poldrack,2018). Neuroendocrine processes are thus likely involved in variabilityof functional brain organization within an individual in a sex-specific manner(Shansky & Murphy,2021)—yet how remains unclear.

In the adult mammalian endocrine system, the production of gonadal steroid hormonesdiffers between the sexes. In females of reproductive age, a major source of dailyvariability in gonadal steroids is dictated by the ovarian cycle, which isresponsible for the cyclical production of estradiol and progesterone over the4–5-day rodent estrous cycle and the monthly human menstrual cycle (Eliot et al., 2023). In humans,both sexes are also subject to cyclical changes in endogenous steroid hormone levelsfollowing the 24-h circadian rhythm, whereby testosterone and cortisol productionpeaks in the morning and steadily declines throughout the day (Dabbs Jr, 1990;Fries et al., 2009). Althoughsteroid hormones are not exclusive to either sex, females generally present higherconcentrations of estrogens and progesterone, and males generally present higherconcentrations of testosterone (Bale& Epperson, 2017), which explains why research primarily focuseson the predominant hormones of each sex accordingly. Despite these substantialdifferences in steroid hormone concentrations between males and females, we lack aformal understanding of how sex-specific neuroendocrine mechanisms may interact withhuman brain organization.

Cross-species evidence points to steroid hormones as potent neuromodulators.Receptors for steroid hormones are expressed throughout the brain, particularly inthe hippocampus and medial temporal lobe (González et al., 2007;Loy et al., 1988;Meffre et al., 2013). Afoundational study in female rats detected a 30% increase in dendritic spine densityof hippocampal CA1 pyramidal neurons on the day of ovulation (peak estradiol)relative to 24 h later (peak progesterone) (Woolley et al., 1990), suggesting estradiol’s rolein enhancing synaptic plasticity in CA1 neurons (Brinton, 2009;Galea et al., 2017;Hao et al., 2003;Hara et al., 2015), while progesterone appears to inhibitthis effect (Woolley & McEwen,1993). Androgens, such as testosterone, also appear to influence medialtemporal lobe morphology, for example, by inhibiting apoptosis in hippocampalneurons (Nguyen et al., 2010).Similarly, findings in humans have linked gonadal steroid levels to changes in brainstructure, for example, through effects of estradiol and progesterone levels onhippocampal morphology over the menstrual cycle (Taylor et al., 2020;Zsido et al., 2023). Studies have also reportedassociations between diurnal steroid hormone fluctuations (including testosterone,estradiol, and cortisol) and total brain volume, gray matter volume, and corticalthickness (Murata et al., 2024),as well as associations between testosterone levels and cortical thickness duringpuberty in regions with high androgen receptor density (Bramen et al., 2012). Moreover, functional magneticresonance imaging (fMRI) studies have revealed associations between human steroidhormone levels and functional brain activity at rest. Studies with samples varyingin size and sampling frequency suggest that changes in functional connectivity inwomen are associated with fluctuating levels of endogenous steroid hormones, such asestradiol and progesterone, over the menstrual cycle (Arélin et al., 2015;Avila-Varela et al., 2024;Hidalgo-Lopez et al., 2021), as well ascontraceptive-dependent levels of exogenous steroid hormones (Engman et al., 2018;Petersen et al., 2014). In men,group analyses have revealed changes in resting-state network connectivity relatedto exogenous increases in testosterone levels (Votinov et al., 2020;Westlye et al., 2017). Although considerable evidencefrom animal and human research supports the role of gonadal steroid hormones inmodulating brain structure and function, whether and how sex-specific endogenousfluctuations in steroid hormones contribute to daily variability in functional brainorganization remains poorly understood.

Gonadal hormones further have the ability to modulate the stress response throughtight interactions between the hypothalamic–pituitary–gonadal (HPG)and hypothalamic–pituitary–adrenal (HPA) axes, which are theneuroendocrine axes, respectively, producing gonadal and adrenal (i.e., cortisol)hormones (Viau, 2002). As such,gonadal steroids are thought to contribute to sex differences in the stress responsethrough their activational and organizational effects on the brain throughout thelifespan (Bale & Epperson,2015). For example, circulating estradiol levels in female rodents appearto elevate cortisol levels during both threatening and non-threatening situations,leading to a more robust HPA axis response relative to male rodents (Oyola & Handa, 2017). Inhumans, estradiol levels have also been shown to modulate healthy female functionalactivity across key regions of the stress circuitry, including the hippocampus,bilateral amygdala, and hypothalamus—an effect that was not observed in womenwith major depressive disorder, suggesting an association between affectivedysfunction and the dysregulation of hormonal effects on stress-related activity(Jacobs et al., 2015). Infact, given that stress contributes to mechanisms of plasticity and vulnerability byphysiologically remodeling neural architecture (McEwen et al., 2015), sex differences in the stressresponse are thought to contribute to differences in the prevalence of affectivepsychiatric disorders (Oyola &Handa, 2017). Moreover, cortisol responsivity seems to both vary (Maki et al., 2015) anddifferentially interact with perceived stress (Duchesne & Pruessner, 2013) at different stages ofthe menstrual cycle, highlighting the importance of also considering effects relatedto subjective self-reported cognitive experience. As such, psychosocial andphysiological stress levels should be considered as potential neurocognitive andneuroendocrine factors affecting dynamic changes in functional brain organizationvia sex-specific mechanisms.

Over the last decade, dense sampling has emerged as a method to investigate thestability and variability of functional brain organization by repeatedly scanningsmaller sets of individuals across longer periods of time. Based on the premise thatnot enough neuroimaging data are collected per individual—yielding estimateswith high measurement error (Poldrack,2021)—recent initiatives such as the MyConnectome Project (Laumann et al., 2015;Poldrack et al., 2015) and theMidnight Scan Club (Gordon et al.,2017) have demonstrated the utility of dense sampling, further inspiringthe collection of several related precision fMRI datasets, reviewed inGratton et al. (2020). Thesestudies revealed fine-grained features unique to the individual, adding a layer ofdetail and specificity that is otherwise overlooked in group-averaged data (Poldrack et al., 2015). Aiming todemonstrate the reliability of resting-state functional connectivity patterns, thesepioneering studies focused on assessing the within-subject stability—ratherthan variability—of the functional connectome (Gratton et al., 2018;Seitzman et al., 2019). As such, they did not investigateco-varying factors and mechanisms that may contribute to day-to-day intra-individualvariability in functional brain activity, nor did they investigate the effects ofsex as a biological variable in their analyses (Shansky & Murphy, 2021). In fact, many densesampling studies so far have focused their analyses on fMRI data without probingunderlying mechanisms or behavioral associations—with some exceptions (e.g.,reporting associations between mood fluctuations and functional connectivitypatterns;Mirchi et al.,2019).

Recently, a dense sampling and deep phenotyping approach has been applied on a23-year-old female (28&Me study;Pritschet et al., 2020) and a 26-year-old male (28&He study;Grotzinger et al., 2024),who were tested over 30 consecutive days in time-locked study sessions includingbrain imaging, venipuncture, salivary sampling, and self-report mood questionnaires.These studies—as well as subsequent studies using the female dataset (e.g.,De Filippi et al., 2021;Fitzgerald et al., 2020;Greenwell et al., 2023;J. M. Mueller et al.,2021)—measured day-to-day changes in functional brain activity,reporting associations between hormonal fluctuations and the reorganization offunctional networks. However, neither of these studies have been used to directlycompare intra-individual variability across sexes, nor have sex-specific researchdesigns been applied to probe and compare neuroendocrine effects in the female andmale subjects in relation to major principles of brain organization, such as the S-Aaxis. In fact, increasing evidence supports the premise of using low-dimensionalmeasures of functional connectivity to study variations in sensory-to-associationhierarchical patterns of intrinsic cortical organization (Bernhardt et al., 2022;Huntenburg et al., 2018;Margulies et al., 2016;Royer et al., 2024). Conceptually,the S-A axis has been shown to reflect both developmental (Sydnor et al., 2021) andevolutionary (Valk et al.,2022;Xu et al., 2020)mechanisms, aligning with microstructural variation (Burt et al., 2018;Paquola & Hong, 2023;Saberi et al., 2023;Valk et al., 2022), as well ascapturing organizational differences between the sexes (Serio et al., 2024).Methodologically, the S-A axis has demonstrated suitable levels of reproducibility,predictive validity, and test–retest reliability (Hong et al., 2020;Knodt et al., 2023). As such,studying daily intra-individual variability along the S-A axis as well as associatedunique neuroendocrine factors in a female and a male would allow to contextualizesubtle intra-individual changes in the functional connectome at a meaningfulorganizational level.

In the current work, we capitalize on a dense sampling approach to investigateintra-individual variability along the S-A axis in two healthy young adults, onemale and one female, from the aforementioned openly available datasets (Grotzinger et al., 2024;Pritschet et al., 2020), probingand comparing both distinct and shared female and male neuroendocrine factors (i.e.,steroid hormone levels), as well as perceived stress, associated with dailyvariability in functional brain organization. We first applied a dimensionalityreduction algorithm to daily functional connectivity matrices in order to computethe S-A axis. After quantifying intra-individual variability along the S-A axis, wedirectly compared patterns of variability between the participants, and furtherdecoded these patterns with publicly available multimodal brain maps. Next, weprobed local- and system-level effects of day-to-day changes in hormone levels andperceived stress on the S-A axis in both participants. Here, we conducted two setsof analyses probing different forms of potential sex specificity by design. First,we specifically assessed effects of steroid hormones that are most predominantwithin each sex (i.e., estradiol and progesterone in the female participant,testosterone in the male participant), as well as cortisol in the male participantgiven its availability and given that its production follows circadian fluctuationpatterns similar to testosterone. Second, we tested for effects of common steroidhormones (i.e., estradiol and testosterone), allowing a direct comparison of effectsacross the female and male participants. As such, rather than systematically testingfor statistical differences between the sexes, our study design capitalizes on sexas a biological variable to investigate particularly relevant as well as commonneuroendocrine factors that may underpin intra-individual variability along a majorprinciple of functional cortical organization in female and male singleindividuals.

## 2 Methods

The current study relies on the use of open data, whose methods have already beenreported elsewhere in detail (seeGrotzinger et al. (2024)andPritschet et al. (2020)for the originalpublications).

### 2.1 Participants and study design

Our sample (*N*= 2) consisted of one female (23 years;data available athttps://openneuro.org/datasets/ds002674/versions/1.0.5;Pritschet et al., 2020) andone male (26 years; data available athttps://openneuro.org/datasets/ds005115/versions/1.0.0;Grotzinger et al., 2024), bothright-handed and Caucasian, with no history of endocrine disorders,neuropsychiatric diagnoses, or head injuries. The female participant reported ahistory of regular menstrual cycles (occurring every 26–28 days onaverage, with no missed periods). As such, through 30 consecutive days of datacollection, the study design and duration aimed to capture the full breadth of amenstrual cycle, in order to capture the full range of possible variation inendogenous estradiol and progesterone levels. Effectively, since the first dayof data collection was not aligned with a specific day or phase of the menstrualcycle, the experimental sessions spanned two cycles. The female participant alsorefrained from taking hormone-based medication in the 12 months preceding datacollection. Participants gave written informed consent for studies that wereoriginally validated by the University of California, Santa Barbara HumanSubjects Committee.

The original study designs for the collection of the female and male dataslightly differed and are fully reported inPritschet et al. (2020)andGrotzinger et al. (2024),respectively. Here, we report the original and complete study designs althoughwe use only part of the collected data for our analyses in order to maximizeconsistency and comparability between the participants (see our data inclusioncriteria below). For 30 consecutive days, both participants underwent behavioralassessments, assessments for hormone analysis (including serological andsalivary assessments), and brain structural and fMRI in time-locked sessions.Experimental sessions for the female participant occurred exclusively inmid-to-late morning, whereas sessions for the male participant took place in theearly morning for the first 10 days, in both the morning and evening for thefollowing 10 days, then exclusively in the late evening for the last 10 days,for a total of 40 sessions, as shown inFigure 1. Due to blood sampling restrictions,the male participant’s serological assessments were conducted in themorning session for the first 15 days and in the evening session for the last 15days, while salivary samples were collected at every session. Each sessionstarted with a behavioral assessment consisting of self-report questionnairesincluding the Perceived Stress Scale (PSS; adapted to reflect past 24 h),consisting of 10 questions measuring the level of appraised stress from lifesituations on a 5-point Likert scale from 0 (“never”) to 4(“very often”), for a total PSS score ranging from 0 (low stress)to 40 (high stress) (Cohen et al.,1983).

**Fig. 1.**
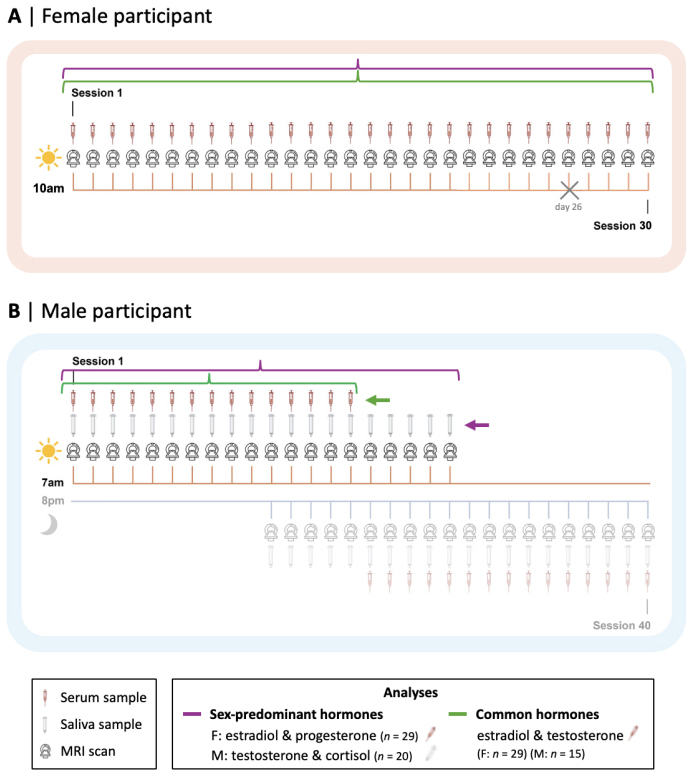
Schematic representation of the experimental designs and analysesinclusion criteria. (A) Female (F) participant; (B) Male (M)participant. For the female participant, experimental day 26 wasexcluded from all analyses due to compromised functional magneticresonance imaging (MRI) data; for the male participant, only morningsession data were analyzed. Figure adapted fromGrotzinger et al.(2024).

The time-locked collection of steroid hormones was conducted in a study-specificmanner. For the female participant, steroid hormone samples were collected viavenipuncture at 10:00 a.m. ± 30 min. For the male participant, salivarysampling and venipuncture were collected at 7 am for morning sessions and at 8pm for evening sessions. Following safety guidelines, blood was drawn only onceon days with two sessions (i.e., in the morning for experimental days11–15 and in the evening for days 16–20). Endocrine samples werecollected after abstaining from food or drink consumption (including caffeineand excluding water) for at least 2 h (female participant), at least 8 h (maleparticipant, morning sessions), and at least 1.5 h (male participant, eveningsessions).

### 2.2 Steroid hormone measurements

For the female participant, serum levels of gonadal steroid hormones(17β-estradiol, progesterone, and testosterone), as well as pituitarygonadotropins (luteinizing hormone (LH) and follicle stimulating hormone (FSH)),were sampled. For the male participant, both serum and salivary levels of totaltestosterone and cortisol were sampled, as well as serum levels of17β-estradiol. The saliva sample (~2 mL) was collected over 5–10min of passive drooling at every session, before storing the sample in a plasticcryovial at -20°C until assayed. Saliva concentrations of testosteroneand cortisol were determined using enzyme immunoassay at the Brigham andWomen’s Hospital Research Assay Core.

For both participants, a 10 cc blood sample was collected per session by alicensed phlebotomist via the insertion of a saline-lock intravenous line intothe dominant or non-dominant forearm and the use of a vacutainer SST (BDDiagnostic Systems). The serum samples were first allowed to clot at roomtemperature for 45–60 min, then centrifuged (2,100 x g for 10 min) andaliquoted into three 2 mL microtubes. The samples were then stored at -20°C until assayed. At the Brigham and Women’s Hospital ResearchAssay Core, liquid chromatography-mass spectrometer (LCMS) was used to determineserum concentration for all steroid hormones, and an immunoassay was used todetermine serum concentration for all gonadotropins in the female participant(i.e., FSH and LH).

Assay sensitivities, dynamic range, and intra-assay coefficients of variation,respectively, were as follows: estradiol, 1 pg/mL, 1–500 pg/mL,<5% relative standard deviation (RSD); progesterone, 0.05 ng/mL,0.05–10 ng/mL, 9.33% RSD; testosterone, 1.0 ng/dL, <4% RSD;testosterone, 1.0 ng/dL, 1–200 ng/dL, <2% RSD; cortisol, 0.5ng/mL, 0.5–250 pg/mL, <8% RSD. Gonadotropin levels were determinedusing chemiluminescent assay (Beckman Coulter), with assay sensitivity, dynamicrange, and the intra-assay coefficient of variation as follows: FSH, 0.2 mIU/mL,0.2–200 mIU/mL, 3.1–4.3%; LH, 0.2 mIU/mL, 0.2–250 mIU/mL,4.3–6.4%.

### 2.3 MRI acquisition

Both participants underwent a 1 h-long MRI scan at every session, conducted on aSiemens 3T Prisma scanner with a 64-channel phased-array head coil. Structuralanatomical images were acquired using a T1-weighted magnetization prepared rapidgradient echo (MPRAGE) sequence (TR = 2,500 ms, TE = 2.31 ms, TI= 934 ms, flip angle = 7°, 0.8 mm thickness) and a gradientecho fieldmap (TR = 758 ms, TE1 = 4.92 ms, TE2 = 7.38 ms,flip angle = 60°). Resting-state fMRI images were acquired with aT2*-weighted multiband echo-planar imaging (EPI) sequence measuring theblood-oxygen-level-dependent (BOLD) contrast (TR = 720 ms, TE = 37ms, flip angle = 52° (female participant) and 56° (maleparticipant), multiband factor = 8; 72 oblique slices, voxel size= 2 mm^3^). The resting-state scans lasted 10 and 15 min for thefemale and male subjects, respectively. To reduce head motion, bothparticipants’ heads were secured in a 3D-printed custom-fitted foam headcase. Overall head motion was minimal for both participants, with a daily meanframewise displacement (FWD) below 130 μm in the female and below 80μm in the male.

### 2.4 fMRI preprocessing

The preprocessing of fMRI data was performed in MATLAB using the StatisticalParametric Mapping 12 (SPM12, Wellcome Trust Centre for Neuroimaging, London)software and is fully reported byPritschet et al. (2020)andGrotzinger et al. (2024). The preprocessing pipelinewas identical for both participants. In short, to correct for head motion andgeometric deformations, functional images were realigned and unwarped, followedby a co-registration of the mean motion-corrected images to the anatomicalimages. The Advanced Normalization Tool’s (ANTs) multivariate templateconstruction was used to normalize all scans to a subject-specific template(Avants et al., 2011).The functional data were subsequently smoothed using a 4 mm full-width halfmaximum (FWHM) isotropic Gaussian kernel. To account for fluctuations in signalintensity across time and space, global signal scaling (median = 1,000)was applied and voxel-wise time series were detrended linearly. After removingthe effects of five sources of physiological noise (cerebrospinal fluid andwhite matter signal) as well as head motion, the residual BOLD signal wasextracted from each voxel. A Volterra expansion of translational/rotationalmotion parameters was used to model head motion based on the Friston-24approach, which accounts for the non-linear and autoregressive effects of headmotion on the BOLD signal (Fristonet al., 1996). In the current study, we did not apply further globalsignal regression.

### 2.5 Functional connectivity and the S-A axis of functional organization

Throughout this work, we used the Schaefer 400-region cortical parcellation(Schaefer et al., 2018)as well as its associated Yeo-Krienen seven functional network solutionincluding the visual, somatomotor, dorsal attention, ventral attention, limbic,frontoparietal, and default-mode networks (Yeo et al., 2011). As reported byPritschet et al. (2020)andGrotzinger et al.(2024), the first eigenvariate across functional volumes was used toextract a regional summary time series in order to compute functionalconnectivity for each scanning session (Friston et al., 2006). Then, using a maximal overlapdiscrete wavelet transform, these regional time series were decomposed intodifferent frequency bands. We used low-frequency fluctuations in wavelets3–6 (~0.01–0.17 Hz) for our subsequent connectivity analyses(Patel & Bullmore,2016). The spectral association between time series data from eachregion was estimated with magnitude-squared coherence, yielding a 400 x 400functional connectivity matrix for each experimental session, indicating thestrength of functional connectivity between all pairs of regions (falsediscovery rate (FDR)-corrected at*q*< 0.05). Coherencewas chosen to measure interregional functional connectivity because it avoidscontamination by physiological noise given that it is not sensitive to the shapeof the regional hemodynamic response function, which can vary as a function ofvascular differences (Sun et al.,2004).

We then applied diffusion map embedding, a non-linear dimensionality reductionalgorithm, on the functional connectivity matrices in order to generatelow-dimensional representations of macroscale functional organization (Margulies et al., 2016).Diffusion map embedding compresses high-dimensional data into low-dimensional“gradients” or axes describing the global structure of the data,along which data points that are highly associated are clustered closer together(i.e., they have similar loadings on the axes), and data points that have lowassociation are further apart (Coifman & Lafon, 2006). To this end, we used the BrainSpacePython toolbox (Vos de Wael etal., 2020) to generate 10 gradients with the following parameters:90% threshold (i.e., only considering the top 10% row-wise z-values offunctional connectivity matrices, representing each seed region’s top 10%of maximally functionally connected regions), α = 0.5 (αcontrols whether the geometry of the set is reflected in the low-dimensionalembedding—i.e., the influence of the sampling points density on themanifold, where α = 0 (maximal influence) and α = 1(no influence)), and t = 0 (t controls the scale of eigenvalues). First,for both participants separately, mean gradients were computed by reducing thedimensionality of their mean functional connectivity matrices (i.e., averagedacross study sessions). Then, using the same parameters, we computed“daily” gradients, that is, for each scanning session. In order tomaintain comparability for intra-individual analyses, the daily gradients werealigned to their respective mean gradients (i.e., per participant) usingProcrustes alignment. Finally, for data from each experimental session, we tookthe well-replicated principal gradient explaining the most variance in the dataand spanning from sensorimotor to association regions (Margulies et al., 2016), whichwe labeled the S-A axis and used to represent functional cortical organization.In our analyses, we refer to S-A axis loadings, which represent each corticalregion’s position on the S-A axis.

### 2.6 Data inclusion

The female subject’s fMRI data collected on experiment day 26 appeared tobe compromised, with the original publication of the dataset reporting that itwas markedly dissimilar to the other study sessions (Pritschet et al., 2020). Wecould confirm this dissimilarity when computing and plotting the S-A axis andcomparing it with the mean S-A axis (averaged across study sessions, excludingday 26),*r*= 0.41,*p*_spin_< 0.001 (seeSupplementary Fig. 1for a visualrepresentation of the female fMRI data on day 26 compared with fMRI dataaveraged across study sessions). There was also a notable difference in thevariance explained in the functional connectivity data by the S-A axis when theS-A axis was computed from the functional connectivity matrix on day 26 (23.7%of variance explained) as opposed to being computed from the mean functionalconnectivity matrix (excluding day 26; 33.95% variance of explained). For thesereasons, we excluded day 26 of the female dataset from our analyses.Furthermore, considering that study designs slightly differed for the twoparticipants, we conducted our analyses on only a part of the data that wereoriginally collected—seeFigure 1for a schematic representation of the experimental designsand analyses inclusion criteria. In our first set of analyses consideringsex-predominant steroid hormones, for the female participant, we chose toinclude serum levels of estradiol and progesterone, as these steroid hormonesare the most potent and studied endocrine neuromodulators in females(*n*= 29). For the male participant, we chose toinclude morning salivary levels of testosterone, as this steroid hormone is amore potent endocrine neuromodulator in males, as well as cortisol given itsavailability and given that its production follows circadian fluctuationpatterns similar to testosterone (*n*= 20). To note,there were some differences in the hormones originally analyzed and available inthe participants’ datasets: Cortisol levels were not provided for thefemale participant and progesterone levels were not provided for the maleparticipant. Furthermore, we chose morning salivary samples for the maleparticipant (rather than serum/evening samples) in this first set of analyses inorder to maximize our sample size (*n*= 20) whilemaintaining intra-individual consistency and keeping the time of data collectioncomparable between participants. Although serum hormone measurements are knownto be more accurate, we confirmed the validity of the salivary hormonemeasurements (and thus their comparability with serum levels) in the maleparticipant by correlating serum and salivary levels for testosterone*(r*= 0.90,*p*= 0.001) andcortisol (*r*= 0.92,*p*= 0.001).In our second set of analyses, aimed at comparing the local- and system-leveleffects of common steroid hormones (i.e., estradiol and testosterone) betweenparticipants, we used morning serum hormone levels for the male participant inorder to increase comparability with the female serum hormone levels (still*n*= 29) at the cost of decreasing male sample size(*n*= 15). We further conducted supplementaryanalyses with reduced female samples (*n*= 20 foranalyses on sex-predominant steroid hormones;*n*= 15 foranalyses on common steroid hormones) to increase comparability with the malesample sizes of*n*= 20 and*n*=15 for the respective analyses. We subsampled the female data points in a mannerthat evenly covered the entire 30-day experimental period (excluding day 26).Specifically, we included the following experimental days: 1, 2, 4, 5, 7, 8, 10,11, 13, 14, 16, 17, 19, 20, 22, 23, 25, 27, 28, 30 (for*n*= 20) and 1, 3, 5, 7, 9, 11, 13, 15, 17, 19, 21, 23, 25, 27, 29 (for*n*= 15).

### 2.7 Statistical analyses

For each participant, intra-individual daily variability in functionalorganization was computed by taking the standard deviation of eachparcel’s S-A axis loading across study sessions. Spearman-rankcorrelation was used to test the similarity of the two participants’intra-individual variability maps, followed by a spin-permutation test with1,000 spherical rotations to control for spatial autocorrelation (Alexander-Bloch et al., 2018).To test for inter-individual differences in intra-individual variability, wefirst quantified differences in variance by subtracting the standard deviationof male S-A axis loadings from the standard deviation of female S-A axisloadings within each cortical region. This subtraction was solely conducted toobtain the directionality of effects, whereby a negative subtraction result in agiven cortical region indicated greater male variance in S-A axis loadingsacross experimental sessions and a positive value conversely indicated greaterfemale variance. Then, to assess the statistical significance of these regionaldifferences in variance between the subjects in each cortical region, we usedthe Levene’s test for equality of variances, which tests the nullhypothesis that the variance of two sets of data is equal. Here, we furtherapplied FDR correction (*q*< 0.05) to control formultiple comparisons across the 400 cortical regions.

To probe other factors that might be associated with intra-individual variabilityin functional organization, we tested, for each participant, the Spearman-rankcorrelation between intra-individual daily variability in S-A axis loadings and19 brain maps from the openly available Neuromaps database (https://github.com/netneurolab/neuromaps;Markello et al., 2022). Weconducted a spin-permutation test with 1,000 spherical rotations for eachcorrelation analysis to control for spatial autocorrelation (Alexander-Bloch et al., 2018),and then applied FDR correction (*q*< 0.05) to controlfor multiple comparisons across the 19 tests conducted per subject. Thefollowing 19 brain maps were selected for their hypothesized relevance tointra-individual variability in S-A axis loadings: The first principal componentof the 123 Neurosynth terms in the Cognitive Atlas, which representsmeta-analytically derived brain functions associated with cortical areas (Yarkoni et al., 2011); thefirst principal component computed for the top 1,000 genes displaying thegreatest variation in expression between cortical gyri of two brains recorded inthe Allen Human Brain Atlas (Hawrylycz et al., 2012); metabolic measures such as glucose, oxygen,and cerebral blood flow (Vaishnaviet al., 2010); receptor densities of dopamine (Alakurtti et al., 2015),acetylcholine (Bedard et al.,2019), serotonin (Beliveau et al., 2017), norepinephrine (Ding et al., 2010), and glutamate (DuBois et al., 2016);structural measures obtained from the Human Connectome Project S1200 release(Van Essen et al.,2013), including group average cortical myelin that was quantified usingMRI T1-weighted/T2-weighted ratio (Glasser et al., 2016) and cortical thickness; electrophysiologicalMEG power distributions from six frequency bands, also obtained from the HumanConnectome Project S1200 release (Van Essen et al., 2013), including alpha (8–12 Hz), beta(15–29 Hz), delta (2–4 Hz), low gamma (30–59 Hz), highgamma (60–90 Hz), and theta (5–7 Hz); and a representation ofevolutionary expansion, based on the cortical surface area expansion frommacaque to human (Hill et al.,2010).

To probe associations between daily changes in brain organization and fluctuatinglevels of steroid hormones and perceived stress, we used linear mixed effectsmodels in complementary local- and system-level approaches. Our local-levelapproach involved testing for local effects (i.e., in each cortical region) ofsteroid hormones and perceived stress on S-A axis loadings, using FDR correctionto control for multiple comparisons of the tested effects across the 400cortical regions (*q*< 0.05). Local-level effects areinformative from a statistical and mathematical perspective, illustrating localshifts in the position of cortical regions on the S-A axis in relation tochanges in steroid hormone and perceived stress levels. Local-level effects alsoallow the statistical comparison of brain-wide patterns of regional effectsacross participants via the Spearman rank correlation of*t*-maps(i.e.,*t*-values across all cortical regions), usingspin-permutation testing with 1,000 spherical rotations to correct for spatialautocorrelation.

Our system-level approach involved investigating effects of steroid hormonelevels and perceived stress on measures of network topology, which describe thephysical organization of nodes in networks and of networks along the S-A axis.For this, we computed measures of within- and between-network dispersion, asdescribed in previous work (Bethlehem et al., 2020;Serio et al., 2024). Within-network dispersion isdefined as the sum of the Euclidean distances squared between network nodes(represented by the parcel S-A axis loadings) to the network centroid(quantified by the median of S-A axis loadings for parcels belonging to the samenetwork), for which a higher value indicates a wider distribution of a givennetwork’s nodes along the S-A axis, indicating greater segregation of thenetwork. Between-network dispersion is defined as the Euclidean distance betweennetwork centroids, for which a higher value indicates that networks are moresegregated from one another along the S-A axis. Within-network dispersion wascomputed for each of the seven Yeo–Krienen functional networks (Yeo et al., 2011), andbetween-network dispersion was computed for each of the 21 possible networkpairs. Then, to test for effects of hormone levels and perceived stress onmeasures of within- and between-network dispersion, we used the same linearmixed effects models that we used to test for local effects. In order to assessstatistical significance, we corrected for multiple comparisons, atBonferroni-corrected thresholds of*p*< 0.004 (0.025/7)for the within-network effects and*p*< 0.001 (0.025/21)for the between-network effects. For effects that survived Bonferronicorrection, we further tested for their spatial specificity. Specifically, foreach model, we generated a null distribution of*t*-values forthe given effect using spin permutation testing (1,000 spherical permutations)of the Schaefer 400 parcellation scheme, thus shuffling the network labels(Alexander-Bloch et al.,2018). We thus controlled for spatial autocorrelation by assessingour empirical*t*-values against our generated nulldistributions, with a significance threshold of*p*_spin_< 0.05. Although system-leveleffects can only be qualitatively compared between participants, they arebiologically informative and interpretable, capturing associations betweenhormone levels and changes in network topology, namely changes in integrationand segregation within and between functional networks.

In order to account for the longitudinal structure of our data (i.e., singlesubject data collected over consecutive days of testing), we used theabove-mentioned linear mixed effects models including “experimentalsessions” as a random effect to capture associations within repeatedmeasures without assuming independence between observations. We also considereddifferent sets of hormones as covariates in our linear mixed effects models, forboth the local- and system-level approaches, in two sets of analyses. Our firstset of analyses aimed to test effects of sex-predominant steroid hormones. Assuch, estradiol and progesterone levels were included as covariates in the modeltesting hormonal effects in the female participant (seeSupplementaryMaterials, Formula 1), and testosterone and cortisol levels wereincluded as covariates in the model testing hormonal effects in the maleparticipant (seeSupplementary Materials, Formula 2).Separate models were used to account for the local effects of perceived stress(PSS score; seeSupplementary Materials, Formula 3). This decision was made*a priori*on the assumption that perceived stress may covarywith the different steroid hormone levels tested in the female and male subjectsto different degrees depending on the hormone (seeSupplementaryFig. 2for correlations between PSS scores and steroid hormone levelsin both subjects). This may lead to varying levels of shared variance betweenperceived stress and steroid hormones and, consequently, including perceivedstress in the main models may differentially impact both the resulting hormonaland perceived stress effects. We, therefore, opted for running independentmodels separating effects by modality (steroid hormones levels vs. self-reportedperceived stress) in our main analyses, in order to minimize bias and increasethe comparability of effects between the participants. We nevertheless conductedsupplementary analyses to show effects yielded by models including both steroidhormones and perceived stress compared with effects yielded by our main,separate models. Our second set of analyses aimed to test effects of commonsteroid hormones (i.e., estradiol and testosterone) in both participants (seeSupplementary Materials, Formula 4). As such, estradiol andtestosterone were included as covariates in the models tested for bothparticipants.

## 3 Results

### 3.1 Daily variability in steroid hormone levels and perceived stress

**Fig. 2.**
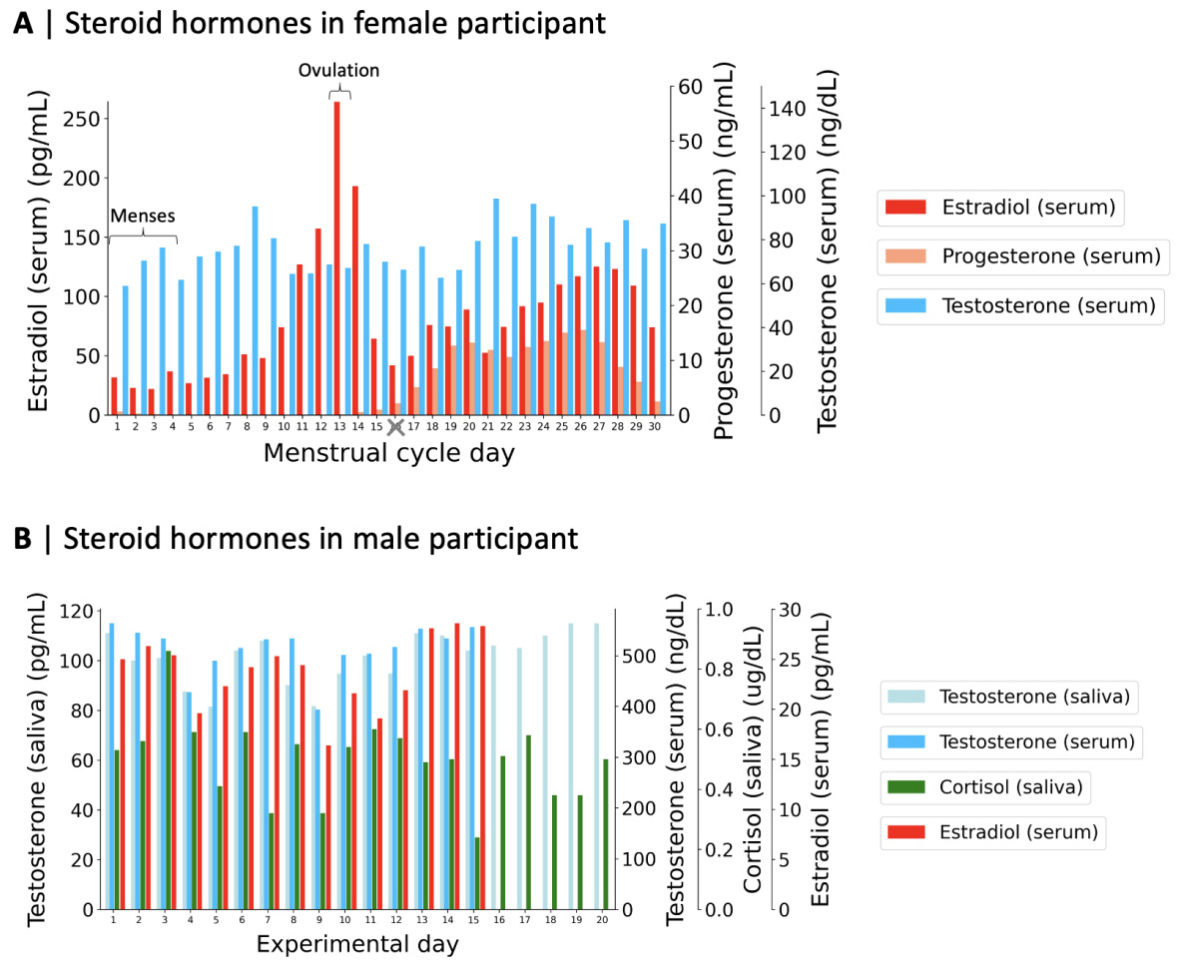
Daily variability in steroid hormone levels. (A) Estradiol, progesterone,and testosterone serum levels in the female participant, capturing thefull spectrum of hormonal variation across the menstrual cycle(*n*= 29), as originally reported byPritschet et al.(2020). Note that the menstrual cycle days shown here do notcorrespond to the experimental sessions, which were rearranged to beginat menstruation for this visualization. Experimental day 26(corresponding to menstrual cycle day 16) was excluded from allanalyses, see Methods for more detail on our data inclusion criteria;(B) Total testosterone and cortisol salivary levels across experimentaldays (*n*= 20), as well as estradiol andtestosterone serum levels across experimental days (*n*= 15) in the male participant as originally reported byGrotzinger et al.(2024).

In the female participant (*n*= 29; we excludedexperimental day 26 from all analyses given that the fMRI data collected on thatday appeared to be compromised—as further reported in our Methods datainclusion criteria), daily serum steroid hormone fluctuations followed expectedpatterns throughout the menstrual cycle (Fig. 2A). Estradiol levels (mean = 84.26± 53.74 pg/mL) showed typical increases and decreases, peaking on day 13of the menstrual cycle (corresponding to the ovulatory window), whileprogesterone levels (mean = 5.25 ± 5.84 ng/mL) were low during thefollicular phase (before ovulation) and high during the luteal phase (afterovulation). Fluctuations in serum testosterone levels (mean = 76.72± 10.51 ng/dL) did not follow any particular or expected pattern. Thesteroid hormone levels of the female subject have been previously reportedelsewhere (Pritschet et al.,2020). In the male participant, fluctuating levels of waking salivarysteroid hormones (*n*= 20), that is, testosterone (mean= 101.61 ± 9.98 pg/mL) and cortisol (mean = 0.50 ±0.13 ug/dL), as well as daily serum steroid hormones (*n*= 15), that is, testosterone (mean = 513.4 ± 45.4 ng/dL)and estradiol (mean = 23.77 ± 3.46 pg/mL), did not follow anyparticular or expected pattern between morning sessions (Fig. 2B). Evening experimental sessions allowedto confirm normative circadian patterns of higher testosterone and cortisollevels in the morning relative to the evening in the male participant, althoughwe excluded data acquired during evening sessions to control for time of day inour analyses (see Methods for more detail on our data inclusion criteria). Thesteroid hormone levels of the male participant have been previously reportedelsewhere (Grotzinger et al.,2024).

Self-reported perceived stress was measured with the perceived stress scale(PSS), where PSS scores can range from 0 (low stress) to 40 (high stress). Wefound no statistically significant difference in PSS scores between the female(mean score = 8.28 ± 6.59) and the male (mean score = 10.10± 2.10) participants, as measured by the Mann–Whitney U Test,*U*= 211.5,*p*= 0.11.

### 3.2 Intra- and inter-individual daily variability in functional cortical organization

**Fig. 3.**
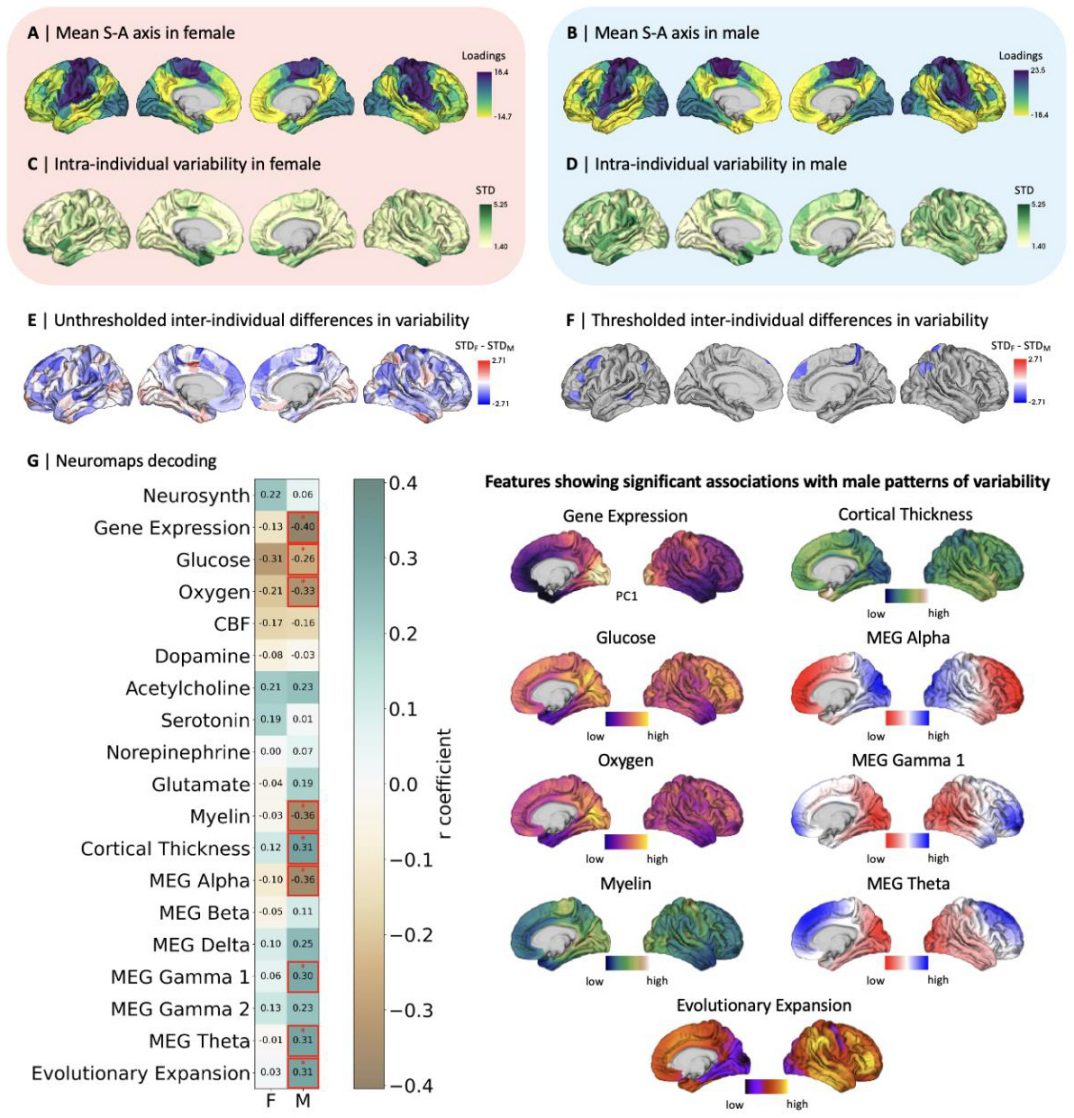
Intra- and inter-individual daily variability in functional corticalorganization. (A) Mean sensorimotor-association (S-A) axis loadingsacross 29 days in the female participant; (B) Mean S-A axis loadingsacross 20 days in the male participant; (C) Intra-individual variabilityin S-A axis loadings quantified by standard deviation (STD) in thefemale participant; (D) Intra-individual variability in S-A axisloadings quantified by STD in the male participant; (E) Inter-individualdifferences in intra-individual variability quantified by thesubtraction of male from female intra-individual variability; (F)Thresholded inter-individual differences in intra-individualvariability, displaying inter-individual difference in intra-individualvariability in false discovery rate (FDR)-corrected parcels(*q*< 0.05) showing statistically significantdifferences as resulted by the Levene’s test for equality ofvariances, namely in about 5% of cortical regions (19 out of 400); (G)Spearman-rank correlations between patterns of intra-individualvariability in the female (F) and male (M) participants and 19 brainfeature maps sourced from the Neuromaps database, where red * andboxes indicate statistically significant correlations after spinpermutation testing and FDR correction (*q*<0.05). Brain feature maps showing statistically significant associationswith the male participant’s intra-individual variability aredisplayed. MEG, magnetoencephalography.

We computed the S-A axis as our measure of functional cortical organization ateach study session in both subjects. For this, we used diffusion map embedding,a non-linear data reduction algorithm, to reduce the dimensionality of the 400 x400 functional connectivity matrices, representing the pairwise strength offunctional connectivity between Schaefer 400 cortical regions (Schaefer et al., 2018). Wethus computed, for each study session within both subjects, the well-replicatedprincipal gradient explaining the most variance in the data (33.95% in thefemale participant, 35.47% in the male participant)—spanning fromunimodal sensorimotor regions to transmodal association regions (Margulies et al.,2016)—which we defined as the S-A axis.Figure 3A and Bshow the mean S-A axes of thefemale and male participants, respectively, computed by applying diffusion mapembedding to the mean daily functional connectivity matrices (averaged acrossstudy sessions) within each participant. We used the S-A axis to representfunctional cortical organization throughout our analyses, where S-A axisloadings represent each of 400 cortical regions’ positions along thislow-dimensional axis of functional cortical organization.

We then probed subtle daily changes in functional cortical organization. For bothparticipants, intra-individual daily variability in S-A axis loadings wasquantified using standard deviation (Fig. 3C, D). We found a statistically significant spatialassociation between the cortex-wide patterns of female and male intra-individualvariability (Spearman’s rank:*r*= 0.29,*p*_spin_< 0.001). Both participantsdisplayed greatest variability in temporal limbic and ventral prefrontalregions, extending further across the cortex in the male participant. Toquantify inter-individual differences in intra-individual variability, wesubtracted male from female standard deviations by cortical parcel (Fig. 3E). We then assessed thestatistical significance of the inter-individual differences across corticalregions with Levene’s test for equality of variances and foundstatistically significant greater local intra-individual variability exclusivelyin the male participant, namely in about 5% of cortical regions (19 out of 400)distributed across functional networks (Fig. 3F). Given the discrepancies in samplesizes for the female (*n*= 29) and male(*n*= 20) participants, we conducted supplementaryanalyses with a reduced female sample of*n*= 20 dailymeasurements. InSupplementary Figure 3C, we show thatpatterns of daily variability in the S-A axis of the female reduced sample aresimilar to those yielded by the full female sample (Fig. 3C),*r*= 0.80,*p*_spin_< 0.001. With comparable female andmale sample sizes, a greater number of cortical regions passed the significancethreshold for inter-individual differences in intra-individual variability,namely 25% of cortical regions (100 out of 400), as illustrated inSupplementaryFigure 3F.

To further interpret intra-individual functional variability, we explored itsassociation with brain features such as gene expression, meta-analyticfunctional activations subserving behavior and cognition, metabolism,neurotransmitter receptor distribution, brain structure and function(electromagnetic waves), as well as patterns of evolutionary expansion. We thusdecoded patterns of intra-individual variability in S-A axis loadings by testingtheir associations with 19 independent maps of brain features from the publiclyavailable Neuromaps database (Markello et al., 2022)—see Methods for more information abouteach map. For this, we computed the Spearman-rank correlation between each brainfeature map and both the female and male intra-individual variability mapsseparately (Fig. 3G). Here, weonly found statistically significant associations that survived spin permutationtesting as well as false discovery rate correction (FDR;*q*< 0.05) for the male participant. Specifically, patterns of maleintra-individual variability were negatively associated with patterns of overallgene expression, glucose and oxygen metabolism, myelin, andmagnetoencephalography (MEG) alpha activity, illustrating that regions withhigher variability are regions typically displaying lower metabolism, myelinintensity, and MEG alpha activity. To note, the directionality of theassociation with gene expression patterns is irrelevant given that the geneexpression map represents the first principal component computed over theexpression of 1,000 genes from the Allen Human Brain Atlas (Hawrylycz et al., 2012), whichshould be understood as an axis of variability in the similarity of geneexpression profiles rather than a measure with meaningful directionality.Furthermore, patterns of male intra-individual variability were positivelyassociated with patterns of cortical thickness, MEG gamma 1 activity, MEG thetaactivity, and evolutionary expansion, illustrating that regions with highervariability are regions typically displaying greater cortical thickness, MEGgamma 1 and theta activity, and greater relative cortical surface area expansionfrom macaque to human. Again, we conducted supplementary analyses to decodepatterns of intra-individual variability in S-A axis loadings for a reducedfemale sample (*n*= 20) and still found no statisticallysignificant associations between female daily variability and the tested brainfeatures (Supplementary Fig. 3G).

### 3.3 Effects of sex-predominant steroid hormones and perceived stress on functional cortical organization

**Fig. 4.**
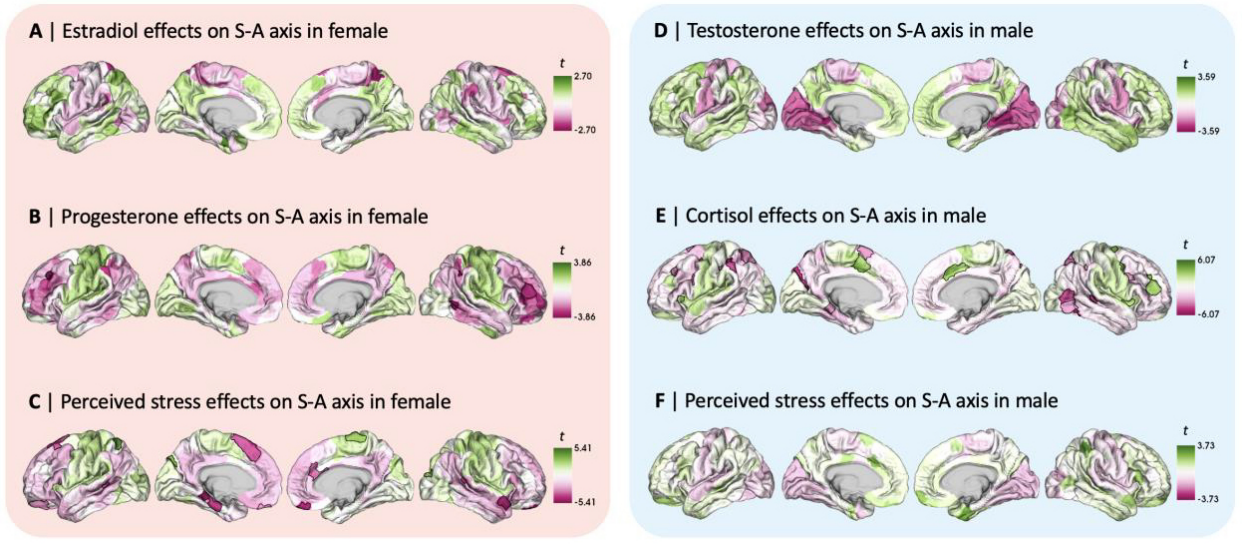
Local-level effects on sensorimotor-association (S-A) axis loadings inthe female and male participants. Unthresholded*t*-mapsof linear mixed effects model results showing patterns of local effectsof (A) Estradiol, (B) Progesterone, and (C) Perceived stress on S-A axisloadings in the female participant; unthresholded*t*-maps of linear mixed effects model results showingpatterns of local effects of (D) Testosterone, (E) Cortisol, and (F)Perceived stress on S-A axis loadings in the male participant.Delineated cortical regions show statistically significant effectsfollowing false discovery rate (FDR) correction (*q*< 0.05), which was used to control for multiple comparisonsacross the 400 cortical regions.

In order to investigate factors potentially underlying dynamic intra-individualdaily changes in functional organization, we tested for local effects of steroidhormone levels and perceived stress on the S-A axis loadings by independentlyfitting different linear mixed effects models in both participants, includingthe random effect of experimental sessions to model the longitudinal structureof our data. Our first set of analyses included steroid hormones that are mostpredominant within each sex (i.e., estradiol and progesterone in the femaleparticipant, testosterone in the male participant), as well as cortisol in themale participant given its availability and given that its production followscircadian fluctuation patterns similar to testosterone. We thus included serumestradiol and progesterone levels as covariates in the model for the femaleparticipant (*n*= 29), and included salivary testosteroneand cortisol levels as covariates in the model for the male participant(*n*= 20). Separate additional models were used toaccount for the local effects of perceived stress (PSS score) in bothparticipants independently. We applied statistical corrections for multiplecomparisons across the 400 cortical regions for each tested effect, using FDRcorrection (*q*< 0.05).*t*-maps of testedlocal effects are displayed inFigure4, where a positive*t*-value denotes a positiveassociation between hormone levels and S-A axis loadings, and a negative t-valueconversely denotes a negative association. In the female participant, whileestradiol did not show statistically significant local-level effects on S-A axisloadings (Fig. 4A),progesterone showed statistically significant effects in 1.3% of corticalregions (5 out of 400;Fig.4B), and perceived stress showed statistically significant effects in 5%of cortical regions (20 out of 400;Fig. 4C). In the male participant, while testosterone (Fig. 4D) and perceived stressed(Fig. 4F) showed nostatistically significant local-level effects on S-A axis loadings, cortisolshowed statistically significant effects in 9.3% of cortical regions (37 out of400;Fig. 4E). Furthermore,the female and male participants showed overall different patterns of perceivedstress effects on functional organization, as indicated by the negativeassociation between female and male spatial patterns of PSS score effects on S-Aaxis loadings,*r*= -0.50,*p*_spin_= 0.001. SeeSupplementaryFigure 4for comparisons of the local-level results reported inFigure 4(independently testingeffects of steroid hormone levels and perceived stress in separate models) andlocal-level results yielded by models including both steroid hormone levels andperceived stress as covariates in the same model. InSupplementaryFigure 5, we also show that patterns of local-level effects ofsex-predominant steroid hormones and perceived stress in a reduced female sample(*n*= 20) are similar to those yielded by the fullfemale sample (illustrated inFig.4A–C).

**Fig. 5.**
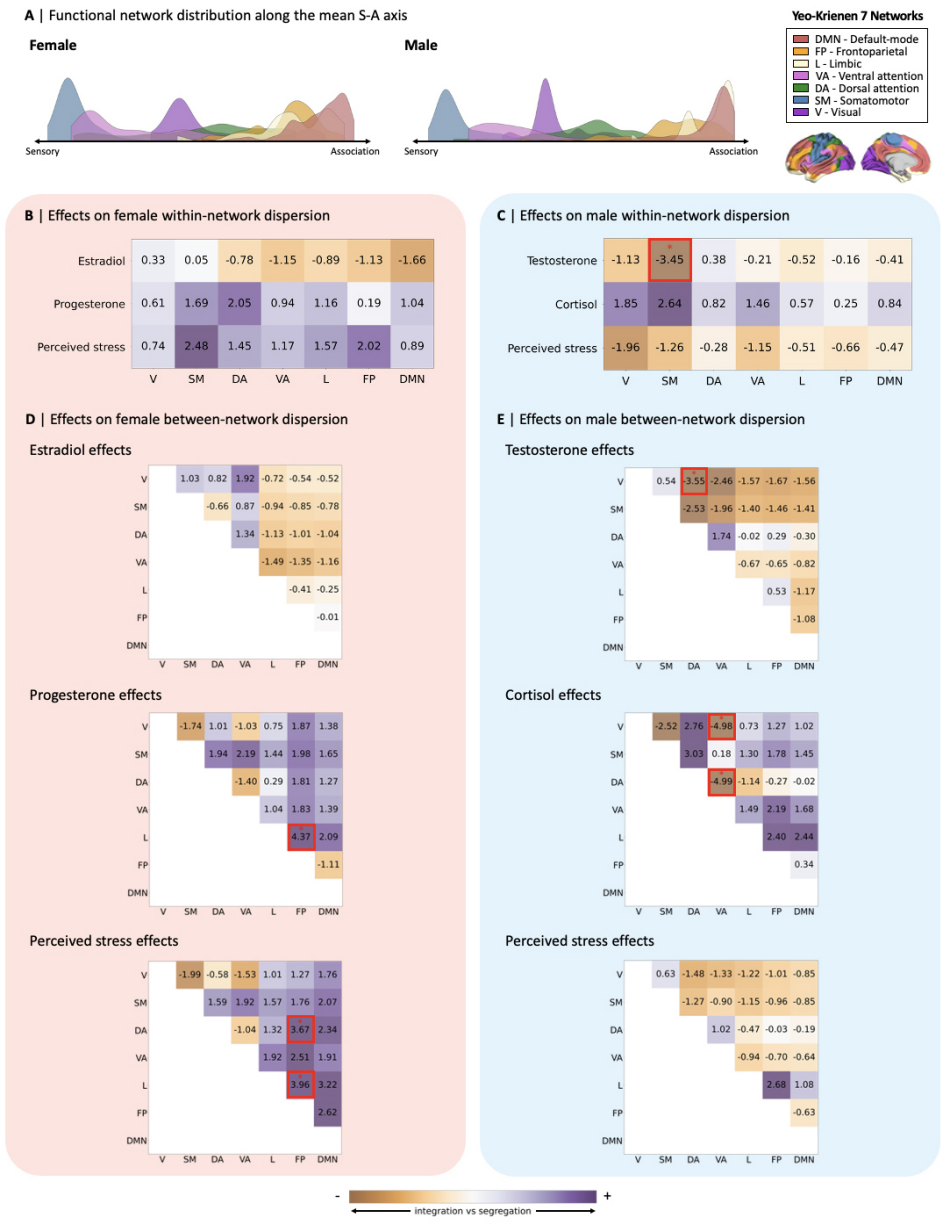
System-level effects of sex-predominant steroid hormones and perceivedstress on functional cortical organization. (A) Visualization of thedistribution of the seven Yeo–Krienen functional networks alongthe mean sensorimotor-association (S-A) axis in the female and maleparticipants. Heatmaps summarizing the*t*-values forsystem-level effects across functional networks of estradiol,progesterone, and perceived stress on the female participant’swithin- (B) and between- (D) network dispersion, and effects oftestosterone, cortisol, and perceived stress on maleparticipant’s within- (C) and between- (E) network dispersion.*t*-values were obtained from linear mixed effectsmodels including different sets of covariates, namely estradiol andprogesterone (for female hormone effects), testosterone and cortisol(for male hormone effects), and perceived stress only (for both femaleand male, tested separately). Red * and boxes indicatestatistical significance of effects corrected for multiple comparisons,at Bonferroni-corrected thresholds of*p*< 0.004(0.025/7) for the within-network dispersion effects and*p*< 0.001 (0.025/21) for the between-networkdispersion effects, as well as corrected for spatial autocorrelation viaspin-permutation testing (1,000 permutations). Positive*t*-values represent higher segregation and negative*t*-values represent higher integration effects. V,visual; SM, somatomotor; DA, dorsal attention; VA, ventral attention; L,limbic; FP, frontoparietal; DMN, default-mode network.

**Fig. 6.**
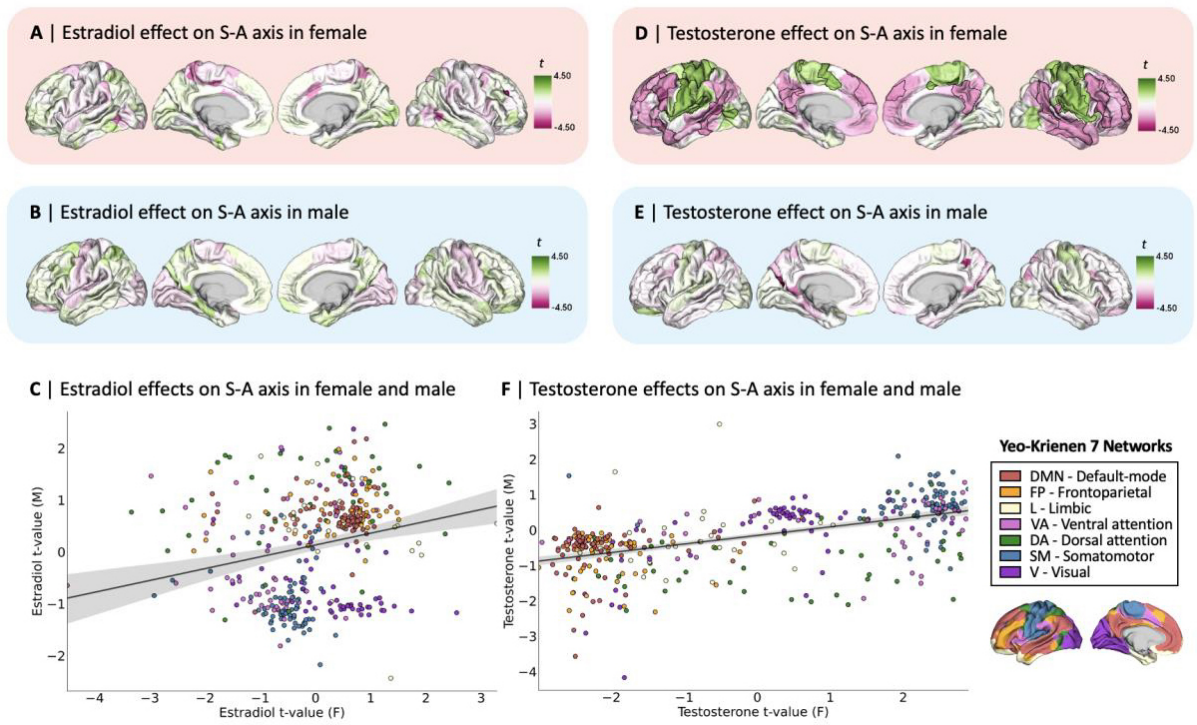
Local-level effects of estradiol and testosterone on functionalorganization in female and male participants. Unthresholded*t*-maps of linear mixed effects model resultsshowing patterns of local effects of estradiol effects on S-A axisloadings in the (A) Female and (B) Male participants. (C) Scatterplotdisplaying the spatial correlation between patterns of local estradioleffects on S-A axis loadings in the female participant (F; x-axis) andin the male participant (M; y-axis),*r*= 0.28,*p*_spin_= 0.003; colors denote theseven Yeo–Krienen functional networks. Unthresholded*t*-maps of linear mixed effects model resultsshowing patterns of local effects of testosterone on S-A axis loadingsin the (D) Female and (E) Male participants. (F) Scatterplot displayingthe spatial correlation between patterns of local testosterone effectson S-A axis loadings in the female participant (x-axis) and in the maleparticipant (y-axis),*r*= 0.57,*p*_spin_= 0.001. Delineatedcortical regions show statistically significant effects following falsediscovery rate (FDR) correction (*q*< 0.05),which was used to control for multiple comparisons across the 400cortical regions.

**Fig. 7.**
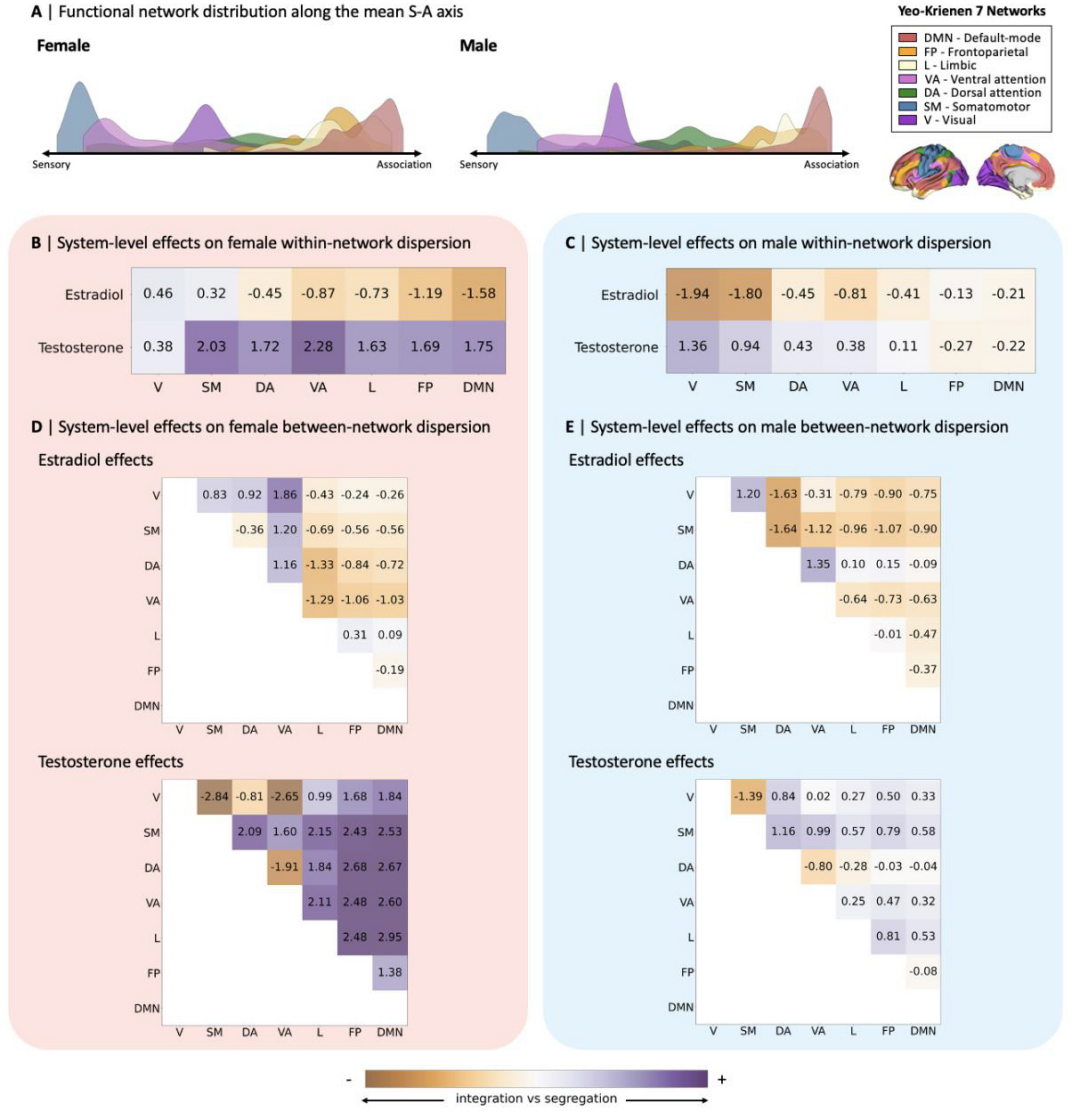
System-level effects of estradiol and testosterone on within- andbetween-network dispersion in female and male participants. (A)Visualization of the distribution of the seven Yeo–Krienenfunctional networks along the mean sensorimotor-association (S-A) axisin the female and male participants. Heatmaps summarizing the*t*-values for system-level effects across functionalnetworks of estradiol and testosterone for the femaleparticipant’s within- (B) and between- (D) network dispersion,and the male participant’s within- (C) and between- (E) networkdispersion.*t*-values were obtained from linear mixedeffects models including estradiol and testosterone as covariates inboth the female and male models, tested separately per participant. Noneof the tested effects were statistically significant after correctionfor multiple comparisons, that is, at Bonferroni-corrected thresholds of*p*< 0.004 (0.025/7) for the within-networkeffects and*p*< 0.001 (0.025/21) for thebetween-network effects. Positive*t*-values representhigher segregation and negative*t*-values representhigher integration effects. V, visual; SM, somatomotor; DA, dorsalattention; VA, ventral attention; L, limbic; FP, frontoparietal; DMN,default-mode network.

For a more interpretable characterization of daily changes in S-A axis loadings(i.e., local shifts in the position of cortical regions along the S-A axis), wetested for system-level effects of hormone levels and perceived stress onchanges in network topology, that is, changes in the topographical organizationof functional networks along the S-A axis (Fig. 5A). For this, we independently computedmeasures of both within- and between-network dispersion across study sessionsfor both participants as done in previous work (Bethlehem et al., 2020;Serio et al., 2024), based on the Yeo-Krienen sevenfunctional network solution (Yeoet al., 2011). Within-network dispersion quantifies the spread ofcortical regions within each of the seven networks along the S-A axis, withhigher values of within-network dispersion indicating higher segregation ofregions within a given network. Between-network dispersion quantifies thepairwise distance between a given pair of functional networks along the S-Aaxis, with higher values of between-network dispersion indicating a highersegregation of the two given networks. We thus computed measures ofwithin-network dispersion for all 7 functional networks, and measures ofbetween-network dispersion for all possible pairwise combinations of the 7networks (i.e., 21 pairs of networks in total). Then, we fitted the same linearmixed effects models used to test for local effects on the S-A axis, separatelytesting for steroid hormone and perceived stress effects on all measures ofwithin- and between-network dispersion. InFigure 5B–E, we summarized patterns ofall tested system-level effects on functional network dispersion along the S-Aaxis, using heatmaps to highlight the directionality of effects, where positive*t*-values are illustrated in purple and negative*t*-values in brown, respectively, indicating segregation andintegration effects. Overall, we found a few statistically significant effects.In the female participant, we found an association between progesterone andincreased segregation between the frontoparietal and limbic networks, as well asassociations between perceived stress and increased segregation of thefrontoparietal network relative to both the limbic and dorsal attention networks(Fig. 5D). In the maleparticipant, we found an association between testosterone and increasedintegration within the somatomotor network (Fig. 5C), as well as associations betweentestosterone and increased integration between the visual and dorsal attentionnetworks, and associations between cortisol and increased integration of theventral attention network relative to both the dorsal attention and the visualnetworks (Fig. 5E). Morebroadly, notable patterns of within-network dispersion effects in the femaleparticipant were that estradiol generally displayed patterns of greaterintegration (particularly within association networks), whereas progesteroneexclusively displayed patterns of greater segregation within all networks (Fig. 5B). In the maleparticipant, testosterone predominantly displayed patterns of greaterintegration within networks, while cortisol was exclusively associated withpatterns of greater within-network segregation (Fig. 5C). Strikingly, effects of perceivedstress showed opposite patterns across the subjects: In the female participant,perceived stress was exclusively associated with increased within-networksegregation, while being exclusively associated with increased within-networkintegration in the male participant. The detailed statistical results for allanalyses of system-level effects on functional organization are summarized inSupplementary Tables 1–3. For further system-level resultsyielded by models including both steroid hormone levels and perceived stress ascovariates, seeSupplementary Figure 6. InSupplementary Figure 7A and C, we also showthat patterns of system-level effects of sex-predominant steroid hormones andperceived stress in a reduced female sample (*n*= 20) aresimilar to those yielded by the full female sample, illustrated inFigure 5B and D.

### 3.4 Effects of common steroid hormones on functional cortical organization

After testing for the hypothesized effects of steroid hormones that are mostpredominant within each sex, our second set of analyses tested for effects ofcommon steroid hormones (i.e., estradiol and testosterone) on functionalorganization in both participants, to allow a direct comparison of effectsacross participants. We thus included estradiol and testosterone as covariatesin a new linear mixed effects model that we independently tested for eachparticipant in order to directly compare the local-level effects of these commonsteroid hormones on S-A axis loadings. To increase comparability betweenparticipants, we used serum hormone levels for the male participant in this setof analyses, at the cost of a smaller sample size (*n*=15; see Methods section for more detail on our data inclusion criteria and theavailability of steroid hormones per subject). As done for the analyses testingfor sex-predominant steroid hormone effects, we applied statistical correctionsfor multiple comparisons across the 400 cortical regions for each tested effect,using FDR correction (*q*< 0.05).*t*-mapsof tested local effects are displayed inFigure 6, where a positive t-value denotes apositive association between hormone levels and S-A axis loadings, and anegative t-value conversely denotes a negative association. Estradiol showedstatistically significant effects on S-A axis loadings in 0.25% of corticalregions (1 out of 400) in the female participant (Fig. 6A) and no statistically significanteffects in the male participant (Fig.6B). The comparison of brain-wide patterns of local effects (i.e.,unthresholded*t*-values) of estradiol in the female and maleparticipants revealed a small statistically significant association(*r*= 0.28,*p*_spin_= 0.003;Fig. 6C).Testosterone showed statistically significant effects on the S-A axis loadingsin 35% of cortical regions (140 out of 400) in the female participant (Fig. 6D) and effects in 0.25% ofcortical regions (1 out of 400) in the male participant (Fig. 6E). The comparison of brain-wide patternsof local effects of testosterone in the female and male participants revealed amedium statistically significant association (*r*= 0.57,*p*_spin_= 0.001;Fig. 6F). InSupplementaryFigure 8, we also show patterns of local-level effects of commonsteroid hormones in a reduced female sample (*n*= 15),which are somewhat weaker for estradiol and somewhat stronger for testosteroneeffects relative to results yielded by the full sample.

For a more interpretable characterization of effects of common steroid hormoneson S-A axis loadings, we tested for system-level effects of estradiol andtestosterone on changes in the topographical organization of functional networksalong the S-A axis (Fig. 7A).InFigure 7B–E, wesummarized patterns of all system-level effects on functional network dispersionalong the S-A axis, using heatmaps to highlight the directionality of effects.Here, we did not find any statistically significant effects of steroid hormoneson within- or between-network dispersion, although patterns of estradiol effectsin the female and testosterone effects in the male remained stable relative toeffects yielded by models including sex predominant hormones. Notably, weobserved diverging patterns effects between the two subjects. For example, inthe male participant, estradiol was associated with greater integration withinsensory networks, opposite to female patterns of estradiol associations withgreater segregation within sensory networks. The detailed statistical resultsfor system-level effects of estradiol and testosterone on functionalorganization in both participants are summarized inSupplementaryTables 4 and 5. InSupplementary Figure 9A and C, we also showpatterns of system-level effects of common steroid hormones in a reduced femalesample (*n*= 15), which are overall somewhat weakerrelative to results yielded by the full sample.

## 4 Discussion

In the current work, we used a dense sampling approach to investigate neuroendocrinefactors that may be associated with intra-individual daily variability in functionalbrain organization in two deeply phenotyped young adults, one female and one male.Different from previous work using dense sampling, we computed a low-dimensionalrepresentation of patterns of resting-state functional connectivity—the S-Aaxis, spanning from unimodal sensorimotor regions to transmodal associationregions—to quantify subtle daily intra-individual variability along this keyhierarchical principle of functional cortical organization and directly comparedvariability across subjects. Overall, participants showed unique cortical patternsof intra-individual variability in S-A axis loadings, with similar cortical areas(i.e., temporal limbic and ventral prefrontal regions) displaying the largest amountof variability across participants and male variability extending further across thecortex. We also found statistically significant greater intra-individual variabilityexclusively in the male participant relative to the female participant, as well asassociations between male whole-brain patterns of intra-individual variability and arange of brain features pertaining to brain metabolism, structure,electrophysiology, genetics, and phylogeny. Our analyses also revealed somestatistically significant local- and system-level effects of steroid hormones andperceived stress on functional organization, which—while demonstrating someshared patterns—also exhibited unique divergences between the female and maleparticipants under study. Collectively, our findings suggest subtle inter-individualdifferences in intra-individual daily variability along a major principle offunctional cortical organization and hint at unique neuroendocrine processes forwhich sex specificity should be further investigated in larger, more diversesamples.

By establishing daily intra-individual variability in the functional corticalorganization, steroid hormone levels, and perceived stress of two densely sampledindividuals (Grotzinger et al.,2024;Pritschet et al.,2020), our findings highlight the dynamic nature of brain function,embedded in equally dynamic endocrine (i.e., steroid hormones) and cognitive systems(i.e., perceived stress) under study. We found that patterns of intra-individualvariability did not follow a particular sensory-to-association differentiation,unlike previous reports of greater within-subject variability in lower orderunimodal regions and greater between-subject variability in higher order transmodalregions (Laumann et al., 2015;S. Mueller et al., 2013).Nevertheless, intra-individual variability was greatest in temporal limbic andventral prefrontal regions in both participants, which to some extent replicatesprevious findings of greater variability in the limbic network of 30 densely sampledindividuals (Chen et al., 2015).Although greater variability in the limbic network may in part reflect the lowersignal-to-noise ratio typically observed in temporal regions during fMRI scans(Arnold Anteraper et al.,2018), limbic regions are also known for their remarkable plasticity,which has been linked to laminar patterns of structural variability (García-Cabezas et al.,2019). The temporal lobe is a cortical area that is particularly dense withsteroid receptors (González etal., 2007;Loy et al.,1988;Meffre et al.,2013), whose volume has been shown to vary as a function of steroidhormone levels (Hoekzema et al.,2017;Taylor et al.,2020;Zeydan et al.,2019;Zsido et al.,2023). Altogether, our findings suggest unique whole-brain patterns ofsubtle daily intra-individual changes along a major principle of functional corticalorganization, with some similarities across participants in the regions displayingthe greatest amount of variability.

Higher variability in S-A axis loadings was exclusively observed in the maleparticipant when statistically testing for inter-individual differences inintra-individual variability along this low-dimensional measure of functionalcortical organization. Given that we only sampled one individual of each sex, wecannot generalize this finding to the group level. Nevertheless, an evolutionaryhypothesis supporting greater male variability in both biological and cognitivephenotypes has long been formulated (Darwin, 1888) and has more recently been empirically supported bydifferent measures of brain structure across the lifespan (Bethlehem et al., 2022;Forde et al., 2020;Wierenga et al., 2022). Greatermale variability is thought to potentially result from constraints imposed bygenetic architecture, namely the heterogametic nature of male sex chromosomes (XY)as opposed to identical sex chromosomes in females (XX) (Reinhold & Engqvist, 2013).In fact, our exploratory analyses showed that intra-individual variability in themale participant was further associated with patterns of overall gene expression, aswell as patterns of glucose and oxygen metabolism, myelin, cortical thickness, MEGalpha, MEG gamma 1, MEG theta activity, and evolutionary expansion. It is importantto note that these different brain features were obtained from openly availabledatasets representing group averages rather than being specific to the individualsunder study. Yet, these multilevel features are theoretically pertinent tofunctional organization and may thus plausibly contribute to intra-individualfunctional variability, as suggested by our findings. For example, metabolicsubstrates such as glucose and oxygen are directly related to the brain’senergy expenditure and local changes in hemodynamics, thus relevant to themeasurement of the blood-oxygen-level-dependent (BOLD) signal (Buxton, 2010). Furthermore, thecomparison of MEG and fMRI signals—that is, local field potentials and theBOLD response, respectively—is conceptually plausible given that bothpredominantly pertain to post-synaptic (dendritic, rather than axonal) signaling(E. L. Hall et al., 2014).Finally, patterns of evolutionary cortical expansion have previously been shown toreflect spatial patterns of variability along the S-A axis (Buckner & Margulies, 2019;Valk et al., 2022;Xu et al., 2020) as well asinter-individual variability in functional connectivity (S. Mueller et al., 2013).Interestingly, the fact that associations between intra-individual variability andthe tested brain features were only statistically significant in the maleparticipant suggests that sources of variability may differ between the twoparticipants. We indeed only observed a low association in patterns ofintra-individual variability between our participants, consistent with previousresearch suggesting that—beyond some shared patterns of variability—alarger proportion of intra-individual daily changes in functional organization isunique to the individual (Laumann etal., 2015;S. Mueller etal., 2013). Our findings thus point to unique multilevel factorsassociated with intra-individual variability, for which sex specificity should befurther investigated in larger samples.

To probe the possible multilevel underpinnings of daily variability along ourlow-dimensional measure of functional cortical organization, we tested both local-and system-level effects of steroid hormone levels and self-reported perceivedstress in the female and male participants separately. Overall, we found someeffects that survived our statistical corrections. At the local level, we observed afew minor effects of steroid hormone levels on S-A axis loadings in bothparticipants, which were spread across functional networks. When comparing effectsof common steroid hormones between the two participants, we observed somesimilarities—but also divergence—in patterns of effects, suggestingsome level of inter-individual differences. Similarly, patterns of local-levelperceived stress effects on the S-A axis loadings were negatively correlated betweenthe participants, and system-level analyses further show that perceived stressshowed patterns of exclusively increased within-network segregation in the femaleparticipant and increased within-network integration in the male participant(although effects did not pass our statistical significance thresholds). This ispossibly in line with previous findings of diverging associations between stress andfunctional connectivity across the sexes, with one study, for example, reporting norelationship between perceived stress and functional connectivity at rest in men asopposed to women (Archer et al.,2018). The literature further points to the possibility of differentstress mechanisms across the sexes, as seen through varying and interacting effectsof cortisol levels and perceived stress in females throughout the menstrual cycle(Duchesne & Pruessner,2013;Maki et al.,2015), as well as associations between resting functional connectivityand both testosterone and cortisol concentrations in males (Ilkevič et al., 2024;Kiem et al., 2013).

System-level analyses further revealed some interestingly diverging patterns ofeffects on network topology across the participants, with statistically significanteffects in the female reflecting greater segregation of more“association” networks, while statistically significant effects inmales exclusively reflected greater integration of more “sensory”networks. Although these findings denote variability within—rather thanbetween—individuals, and are derived from only one subject of each sex, theyare somewhat reminiscent of previously reported sex differences in functionalconnectivity. For instance, the literature has most consistently reported greaterfunctional connectivity in females in association networks such as within regionsbelonging to the DMN (Allen et al.,2011;Biswal et al.,2010;Bluhm et al.,2008) and greater functional connectivity in males within somatomotorregions (Biswal et al., 2010;Scheinost et al., 2015).Our previous work also suggests, from the perspective of connectivity profiles, thatfemales make stronger connections within the DMN and males make stronger connectionsinvolving the somatomotor network (Serio et al., 2024). Furthermore, our findings of diverging patterns ofeffects on network topology between the female and male participants, yielded bymeasures of network dispersion, can be meaningfully interpreted as topologicalchanges in functional communities becoming more similar or different along thehierarchical domains of the S-A axis (Bethlehem et al., 2020;Serio et al., 2024). Functional network integration and segregation aremore generally considered to be important indicators of network topologicalstructure and reconfiguration underpinning cognition, as they have been associatedwith a range of cognitive functions as well as changes in brain states, arousal, andenergy expenditure (Shine &Poldrack, 2018). Altogether, our findings underscore heterogeneous andwidespread patterns of effects that are not specific to a set of regions ornetworks. The divergence of findings between the participants further suggestsindividual differences in neuroendocrine and stress effects on network topology,hinting at processes that may vary across sexes but require systematic andstatistical testing in larger samples.

Despite the insights gained through our study, some limitations should beacknowledged. First, our small sample of two young healthy adults—one female(*n*= 29) and one male (*n*=20/15)—not only entails low statistical power, likely underpinning thescarcity of statistically significant effects in some analyses of our study, butalso precludes the generalization of results. On the one hand, although the studydesign effectively captured a snapshot of the full range of possible endogenousfemale steroid hormone variation across consecutive days dictated by the menstrualcycle, more data would be required for both the female and male participants togeneralize within-subject findings across longer periods of time. On the other hand,larger heterogeneous samples of different and diverse individuals are required togeneralize findings at the population level. We used data that were collected with ahigh sampling frequency, conscious of the trade-off of data depth (i.e., repeateddeep phenotyping in the same individuals) over breadth (i.e., across multipleindividuals). As previously eloquently formulated: “Just as no single brainis representative of a population, no group-averaged brain represents a givenindividual” (Laumann et al.,2015). Our focus was thus not to yield generalizable findings per se, butto probe fine-grained intra-individual effects that provide a multilevel account offactors potentially influencing intra-individual variability in functionalorganization. In fact, recent work from our group has highlighted sex differences infunctional organization in a large sample (*N*= 1,000), whichcould not be explained by differences in cortical morphometry (Serio et al., 2024), requiring adeeper investigation of other potential explanations, such as neuroendocrine andneurocognitive factors. Although our group has also observed sex differences inisocortex and hippocampus microstructure related to self-reported female menstrualcycle stage and hormonal contraceptive use (Küchenhoff et al., 2024), those measures were onlyproxies of female hormone levels, and establishing neuroendocrine mechanismsstrictly requires endocrine samples. Our current study thus allowed us to bridgeboth methodological and conceptual gaps with a deeply phenotyped sample, providinginsights into intra-individual variability that was not otherwise possible in largesamples and thus highlighting the complementary nature of both approaches (Poldrack, 2021).

Second, with respect to interpreting sex effects in our current study, we cannotdetermine the extent to which the observed inter-individual differences inintra-individual variability may be explained by sex-specific mechanisms as opposedto broader individual differences. By only including one individual of either sex,we cannot assume the degree to which our participants may be representative of thegreater female and male populations, respectively, with further increasing evidencesuggesting that sex should actually be treated as a continuous variable inbiological research (Neuhoff,2022;Wiersch &Weis, 2021). Furthermore, there were differences in the available datacollected for the female and the male participants, which both constrained the scopeof our analyses (i.e., progesterone effects only tested in the female and cortisoleffects only tested in the male) and may have further resulted in systematicdifferences in the observed effects (i.e., due to methodological differences in datacollection). However, our data inclusion criteria specifically aimed to minimizedifferences and thus maximize the comparability of effects across participants. Theoutstanding differences in sample size (i.e., more female data points) and hormonesampling method (i.e., saliva vs. serum) should have had minimal impact on theresults, as indicated by our supplementary analyses on reduced female samples, aswell as by the high correlations of testosterone and cortisol levels measured in themale participant’s saliva and serum. In terms of the differences in hormonesfocused on for our female and male subjects, future work exploring the dynamics ofall major steroid hormones across the sexes will yield a stronger understanding ofthe interplay between the endocrine and nervous systems. Another limitation relatedto the categorical conceptualization of biological sex is that we did not considerpossible effects of steroid hormones on functional organization in gender-diverseindividuals who challenge the notion of binary female–male categories. Infact, steroid hormone levels are not fixed and may be dynamically affected bygendered social experiences (Hyde etal., 2019), as well as a range of more general environmental factors thatgo beyond sex and gender identity, such as sleep, nutrition, caffeine consumption,physical exercise, and stress (Chichinadze & Chichinadze, 2008;Glover et al., 2022;D. C. Hall, 2001;Ives et al., 2011;Kraemer & Ratamess, 2005;Lord et al., 2014;Roney & Simmons, 2015;Sisti et al., 2015;Wrzosek et al., 2020). As such, alarger sample capturing greater variability across females, males, and individualsin general is necessary to establish the degree of sex specificity of the effectstested in our study, as well as to further assess possible gender-specific effects(Dhamala et al., 2024). Ourstudy is, however, novel in probing and comparing both distinct and shared femaleand male neuroendocrine effects on the S-A axis in densely sampled individuals.

Third, it could be contended that our findings of intra-individual daily variabilityin functional cortical organization may be capturing random noise in our data.However, we explicitly made methodological decisions aimed at reducing biases andnoise particularly pertaining to daily variability and endocrine effects. First, weused the S-A axis as our measure of functional cortical organization,which—through its low dimensionality and thresholding—has been shownto have greater test–retest reliability than more commonly used measures ofunthresholded edge-wise functional connectivity (Hong et al., 2020;Knodt et al., 2023). In terms of data design, studysessions were time locked, and food and caffeine intake prior to study sessions wasstrictly controlled through abstinence in order to limit confounding physiologicaleffects (Grotzinger et al.,2024;Pritschet et al.,2020). Steroid hormones are also thought to potentially inducephysiological artifacts, such as local changes in cerebral blood perfusion (Ghisleni et al., 2015), whichcould be mistaken as cognitively pertinent changes in brain function (Laumann & Snyder, 2021).However, various steps were taken in the preprocessing of our fMRI data—suchas global signal scaling and linear detrending of voxel-wise time series—toaccount for temporal and spatial fluctuations in signal intensity and to remove theeffects of head motion and physiological noise features such as cerebral spinalfluid from the BOLD signal. We also used coherence as our measure of functionalconnectivity, which is known for its robustness to temporal variability in regionalhemodynamics as well as its measurement of time series covariances in frequenciesoutside the spectrum prone to contamination by physiological noise (Sun et al., 2004). As such, theintra-individual daily changes in variability observed in our study are likely toreflect meaningful fluctuations in signal beyond noise. Nevertheless, more researchis required to assess the directionality of hormonal effects on functional brainorganization, specifically testing causality in statistical relationships andfurther probing mechanistic biological explanations of lagged hormonal effects,which are reported elsewhere (Pritschetet al., 2020) but go beyond the scope of our study.

All in all, by observing subtle daily changes along a low-dimensional measure offunctional cortical organization in two densely sampled healthy young adults,co-occurring with fluctuations in steroid hormone levels and perceived stress, ourfindings underscore the importance of holistically considering the brain as an organembedded in an extensive network of interacting endocrine and psychophysiologicalsystems. By observing diverging patterns of effects in a female and a maleindividual, we highlight the need for research to systematically test for sexeffects, particularly considering the sex specificity of neuroendocrine mechanisms(Shansky & Murphy,2021). Importantly, by showing that a male individual is as subject tohormone-related fluctuations in functional brain organization as a female, we debunkthe deeply rooted belief that endocrine variability is an exclusively femaleconcern, which has led to the historical underrepresentation of women from researchstudies (Jacobs, 2023). Goingforward, giving equal consideration to both sexes—as well as combining densesampling approaches with large population-based studies—is necessary to gaina thorough understanding of neuroendocrine and neurocognitive processes underlyingvariability along principles of functional brain organization in health anddisease.

## Supplementary Material

Supplementary Material

## Data Availability

All data needed to evaluate the conclusions in the paper are present in the paper andthe Supplementary Materials. We obtained data from the open-access 28&Mesample (Pritschet et al., 2020),available athttps://openneuro.org/datasets/ds002674/versions/1.0.5, and the28&He sample (Grotzinger et al.,2024), available athttps://openneuro.org/datasets/ds005115/versions/1.0.0. The code used to conduct the analyses presented in this manuscript is available athttps://github.com/biancaserio/MC_gradients. The code used to preprocessthe data used in this manuscript is available athttps://github.com/tsantander/PritschetSantander2020_NI_Hormones. Thegeneral code and tutorials for functional gradient decomposition can further befound athttps://brainspace.readthedocs.io/en/latest/index.html.

## Supplementary Materials

Supplementary results can be found in the Supplementary Materials. Supplementarymaterial for this article is available here:https://doi.org/10.1162/imag_a_00474.
