# Supplementary material for: Exploring neuroendocrine influences on the sensorimotor-association axis in a female and a male individual
Source: Imaging Neurosci (Camb). 2025 Feb 18;3:imag_a_00474. doi: 10.1162/imag_a_00474 (PMC12319837; doi:10.1162/imag_a_00474)
Supplement: Supplementary Material [file imag_a_00474-supp.pdf]

Supplementary Materials for

Exploring neuroendocrine influences on the sensorimotor-association axis in a female and male individual

Bianca Serio[†*], Deniz Yilmaz[†], Laura Pritschet, Hannah Grotzinger, Emily G. Jacobs, Simon B. Eickhoff, Sofie L. Valk[*]

† Shared first-author

* Correspondence to
Bianca Serio (b.serio@fz-juelich.de)
Sofie L. Valk (valk@cbs.mpg.de)

Supplementary Methods

<u>Formulas for the different linear mixed effects models included in our main analyses</u>

Formulas for which the dependent variable (DV) varies as a function of the analysis. Specifically, for local-level effects, DV = sensorimotor-association (S-A) axis loadings, and for system-level effects, DV = measures of within-/between-network dispersion metric.

Results presented in Figure 4 (local-level effects) and Figure 5 (system-level effects)

Model testing for the effects of sex-specific steroid hormones in the female:

$$\boldsymbol{DV \sim estradiol + progesterone + (1 \mid experimental\ session)} \quad (1)$$

Model testing for the effects of sex-specific steroid hormones in the male:

$$\boldsymbol{DV \sim testosterone + cortisol + (1 \mid experimental\ session)} \quad (2)$$

Model testing for the effects of perceived stress (perceived stress scale (PSS) score) across sexes:

$$\boldsymbol{DV \sim PSS\ score + (1 \mid experimental\ session)} \quad (3)$$

Results presented in Figure 6 (local-level effects) and Figure 7 (system-level effects)

Model testing for the effects of common steroid hormones across sexes:

$$\boldsymbol{DV \sim estradiol + testosterone + (1 \mid experimental\ session)} \quad (4)$$

Supplementary Results

Figures

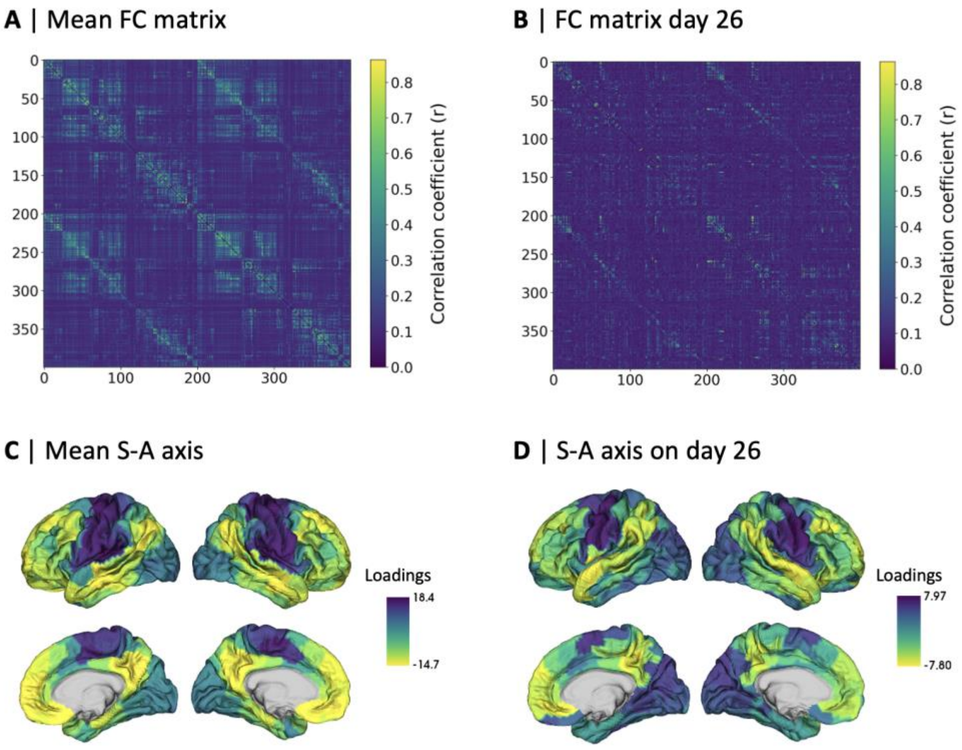

Supplementary Figure 1. Female functional magnetic resonance data on day 26 and averaged across study sessions. A | Mean functional connectivity (FC) matrix, i.e., averaged across experimental sessions excluding day 26 (*n* = 29); **B** | FC matrix on day 26; **C** | Mean sensorimotor-association (S-A) axis, i.e., computed from the mean FC matrix averaged across experimental sessions excluding day 26 (*n* =29); **D** | S-A axis on day 26, i.e., computed from the FC matrix on day 26.

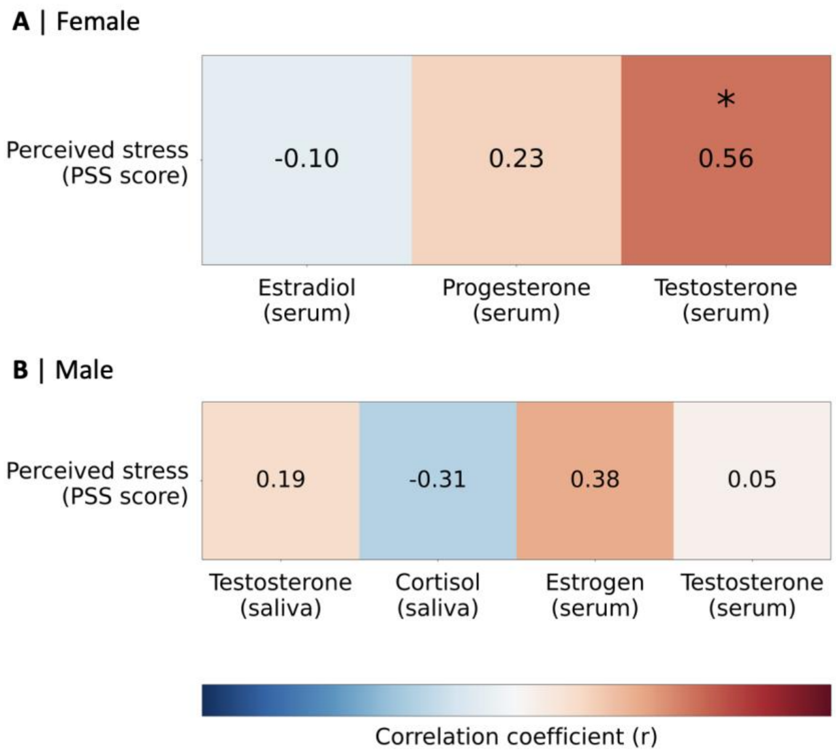

Supplementary Figure 2. Associations between perceived stress and steroid hormone levels by sex. Heatmaps summarizing the Spearman's rank correlation coefficient (*r*) values for the associations between perceived stress (quantified by the perceived stress scale (PSS) score) and steroid hormone levels in the **A** | female and **B** | male participants. * indicates statistical significance of effects at the $p < 0.05$ threshold. The only statistically significant association is between PSS score and testosterone in the female ($r = 0.56$, $p = 0.002$).

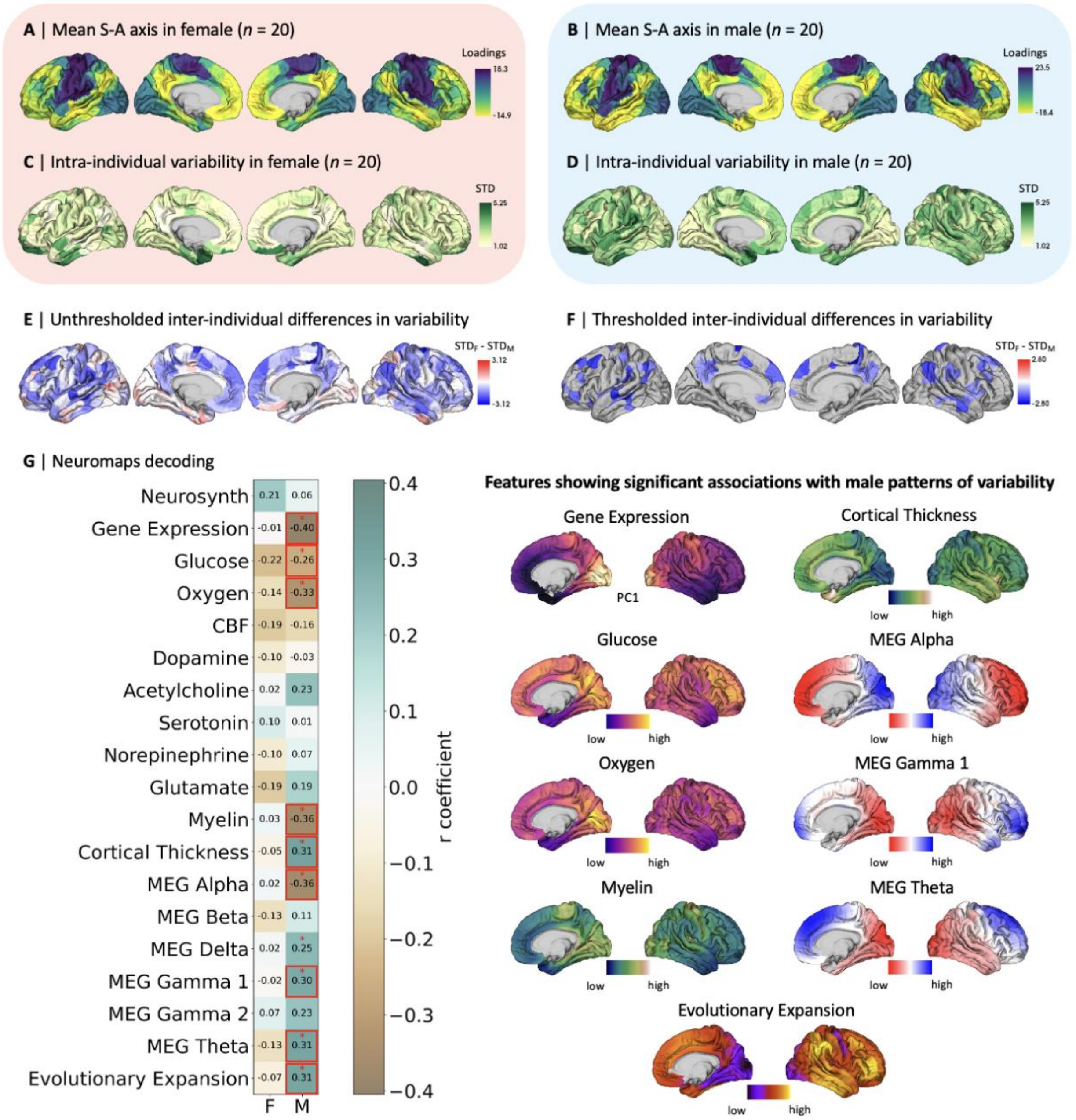

Supplementary Figure 3. Intra- and inter-individual daily variability in functional cortical organization for comparable female and male sample sizes (*n* = 20). **A** | Mean sensorimotor-association (S-A) axis loadings across 20 days in the female participant; **B** | Mean S-A axis loadings across 20 days in the male participant; **C** | Intra-individual variability in S-A axis loadings quantified by standard deviation (STD) in the female participant; **D** | Intra-individual variability in S-A axis loadings quantified by STD in the male participant; **E** | Inter-individual differences in intra-individual variability quantified by the subtraction of male from female intra-individual variability; **F** | Thresholded inter-individual differences in intra-individual variability, displaying inter-individual difference in intra-individual variability in false discovery rate (FDR)-corrected parcels ($q < 0.05$) showing statistically significant differences as resulted by the Levene's test for equality of variances, namely in 25% of cortical regions (100 out of 400); **G** | Spearman-rank correlations between patterns of intra-individual variability in the female (F) and male (M) participants and 19 brain feature maps sourced from the Neuromaps database, where red * and boxes indicate statistically significant correlations after spin permutation testing and FDR correction ($q < 0.05$). Brain feature maps showing statistically significant associations with the male participant's intra-individual variability are displayed. MEG, magnetoencephalography.

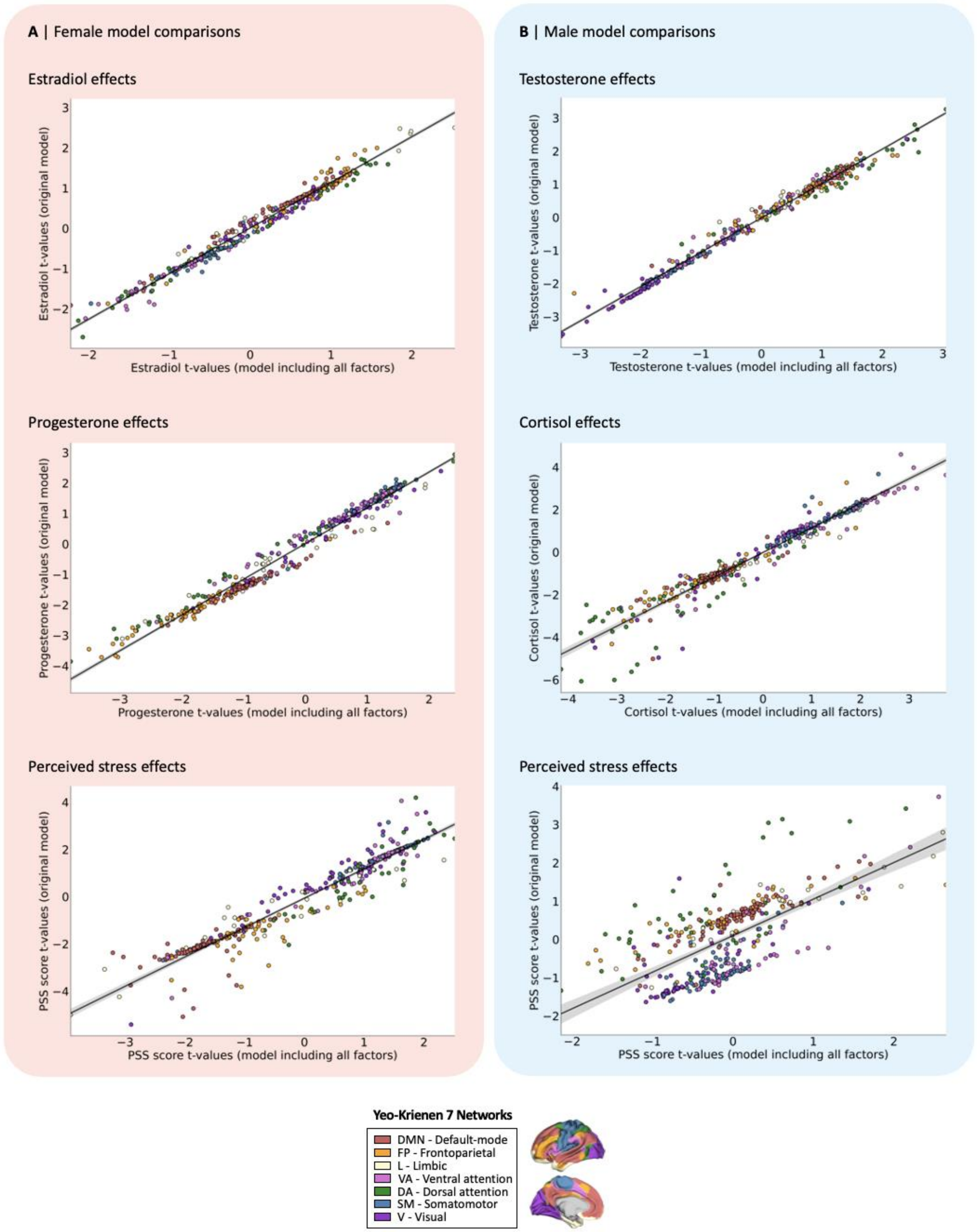

Supplementary Figure 4. Comparison of models computing effects of sex-predominant steroid hormones and perceived stress on functional cortical organization: original models independently including steroid hormones and perceived stress vs models including all covariates by sex. Scatterplots displaying the spatial correlations of local-level effects on the S-A axis loadings by model, namely effects yielded by original models independently

including steroid hormones and perceived stress (PSS score) (y-axis) and models including all factors (x-axis). Comparison of model results in **A |** the female participant: Estradiol, $r = 0.99$, $p_{spin} < .001$; progesterone, $r = 0.98$, $p_{spin} = .001$; PSS, $r = 0.96$, $p_{spin} < .001$. Comparison of model results in **B** | the male participant: Testosterone, $r = 0.99$, $p_{spin} < .001$; cortisol, $r = 0.98$, $p_{spin} < .001$; PSS, $r = 0.68$, $p_{spin} < .001$. Colors denote the seven Yeo-Krienen functional networks.

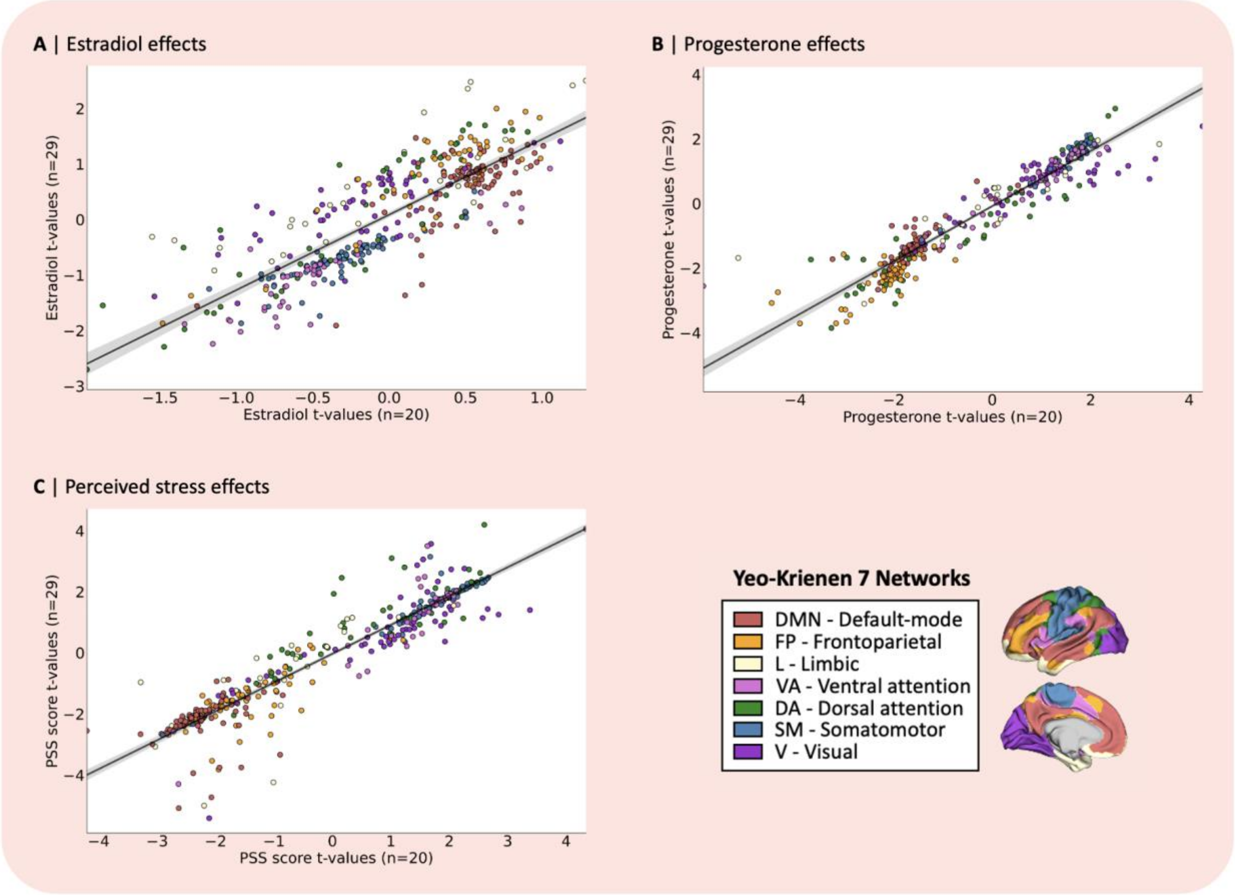

Supplementary Figure 5. Comparison of female effects of sex-predominant steroid hormones and perceived stress on functional cortical organization depending on sample size: full sample (*n* = 29) vs reduced sample (*n* = 20). Scatterplots displaying the spatial correlations of local-level effects on the S-A axis loadings by sample size, namely effects yielded by full sample (*n* = 29; y-axis) and reduced sample (*n* = 20; x-axis). Comparison of effects for **A |** Estradiol, $r = 0.83$, $p_{spin} < .001$; **B** | Progesterone: $r = 0.96$, $p_{spin} < .001$; **C** | Perceived stress (PSS score): $r = 0.93$, $p_{spin} < .001$. Colors denote the seven Yeo-Krienen functional networks.

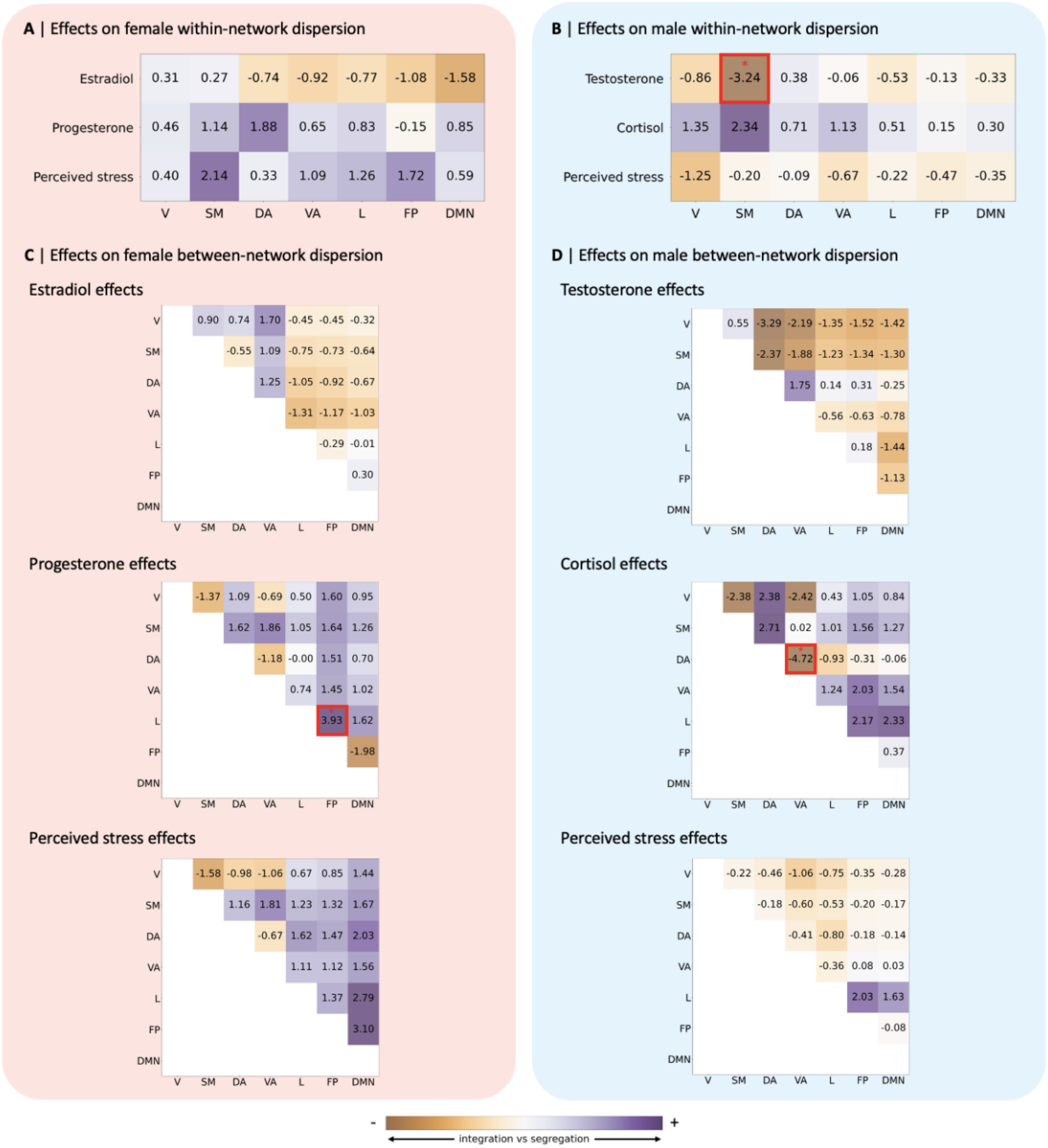

Supplementary Figure 6. System-level effects of sex-predominant steroid hormones and perceived stress on functional cortical organization in models including all covariates by sex (as opposed to using separate models for steroid hormones and for perceived stress). Heatmaps summarizing the *t*-values for system-level effects across functional networks of estradiol, progesterone, and perceived stress on the female participant's **A** | within- and **C** | between- network dispersion, as well as effects of testosterone, cortisol, and perceived stress on male participant's **B** | within- and **D** | between- network dispersion. *t*-values were obtained from linear mixed effects models including different sets of covariates by sex, namely estradiol, progesterone, and perceived stress (for female effects), and testosterone, cortisol, and perceived stress (for male effects). Red * and boxes indicate statistical significance of effects corrected for multiple comparisons, at Bonferroni-corrected thresholds of $p < 0.004$ (0.025/7) for the within-network dispersion effects and $p < 0.001$ (0.025/21) for the between-network dispersion effects, as well as corrected for spatial autocorrelation via spin-permutation testing (1000 permutations). Positive *t*-values represent higher segregation and

negative *t*-values represent higher integration effects. V, visual; SM, somatomotor; DA, dorsal attention; VA, ventral attention; L, limbic; FP, frontoparietal; DMN, default-mode network.

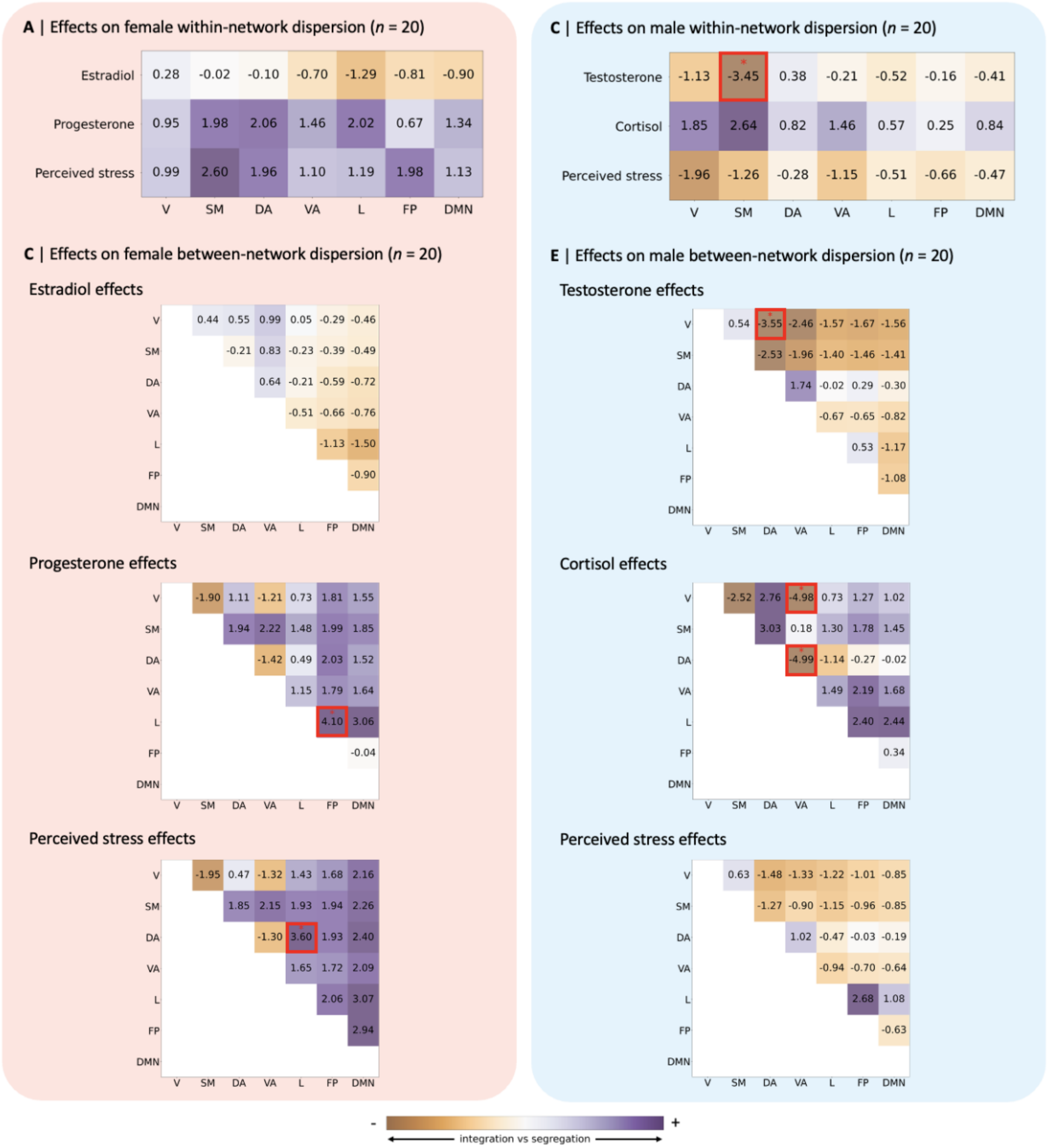

Supplementary Figure 7. System-level effects of sex-predominant steroid hormones and perceived stress on functional cortical organization for comparable female and male sample sizes (*n* = 20). For both sexes with equal sample sizes (*n* = 20), effects of sex-predominant steroid hormones and perceived stress were tested in linear mixed effects models. Note that only the female results are novel (reduced sample) – the male results correspond to those reported in the main analyses. Heatmaps summarizing the *t*-values for system-level effects across functional networks of estradiol, progesterone, and perceived stress on the female participant's within- (**A**) and between- (**C**) network dispersion, and effects of testosterone, cortisol, and perceived stress on male participant's within- (**B**) and between- (**D**) network dispersion. *t*-values were obtained from linear mixed effects models including different sets of covariates, namely estradiol and progesterone (for female hormone effects), testosterone and cortisol (for male hormone effects), and perceived stress only (for both female and male, tested separately). Red * and boxes indicate statistical significance of effects corrected for multiple comparisons, at Bonferroni-corrected thresholds of $p < 0.004$ (0.025/7) for the within-

network dispersion effects and $p < 0.001$ (0.025/21) for the between-network dispersion effects, as well as corrected for spatial autocorrelation via spin-permutation testing (1000 permutations). Positive *t*-values represent higher segregation and negative *t*-values represent higher integration effects. V, visual; SM, somatomotor; DA, dorsal attention; VA, ventral attention; L, limbic; FP, frontoparietal; DMN, default-mode network.

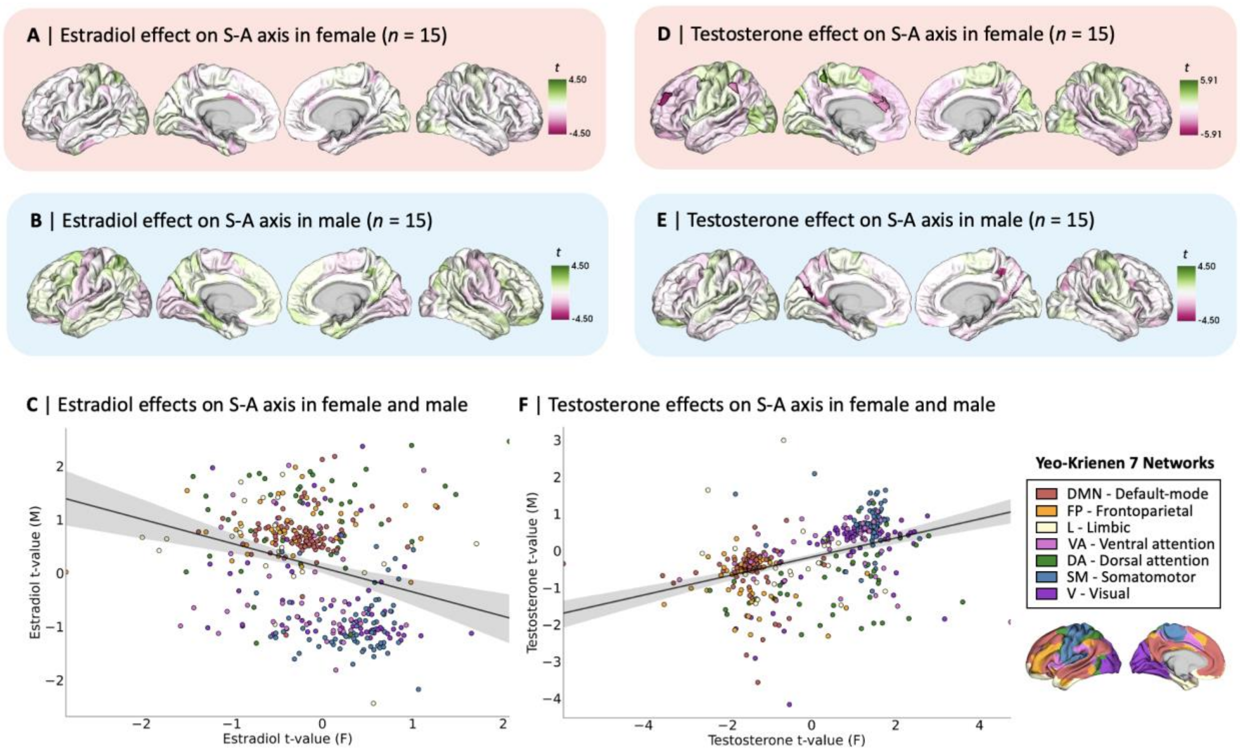

Supplementary Figure 8. Local-level effects of estradiol and testosterone on functional organization for comparable female and male sample sizes (*n* = 15). For both sexes with equal sample sizes ($n = 15$), effects were tested in linear mixed effects models including estradiol and testosterone as covariates. Note that only the female results are novel (reduced sample) – the male results correspond to those reported in the main analyses. Unthresholded *t*-maps of linear mixed effects model results showing patterns of local effects of estradiol effects on S-A axis loadings in the **A** | Female and **B** | Male participants. **C** | Scatterplot displaying the spatial correlation between patterns of local estradiol effects on S-A axis loadings in female participant (F; x-axis) and in male participant (M; y-axis), $r = -0.34$, $p_{spin} = 0.003$; colors denote the seven Yeo-Krienen functional networks. Unthresholded *t*-maps of linear mixed effects model results showing patterns of local effects of testosterone on S-A axis loadings in the **D** | Female and **E** | Male participants. **F** | Scatterplot displaying the spatial correlation between patterns of local testosterone effects on S-A axis loadings in female participant (x-axis) and in male participant (y-axis), $r = 0.51$, $p_{spin} = 0.001$. Delineated cortical regions show statistically significant effects following false discovery rate (FDR)-correction ($q < 0.05$), which was used to control for multiple comparisons across the 400 cortical regions.

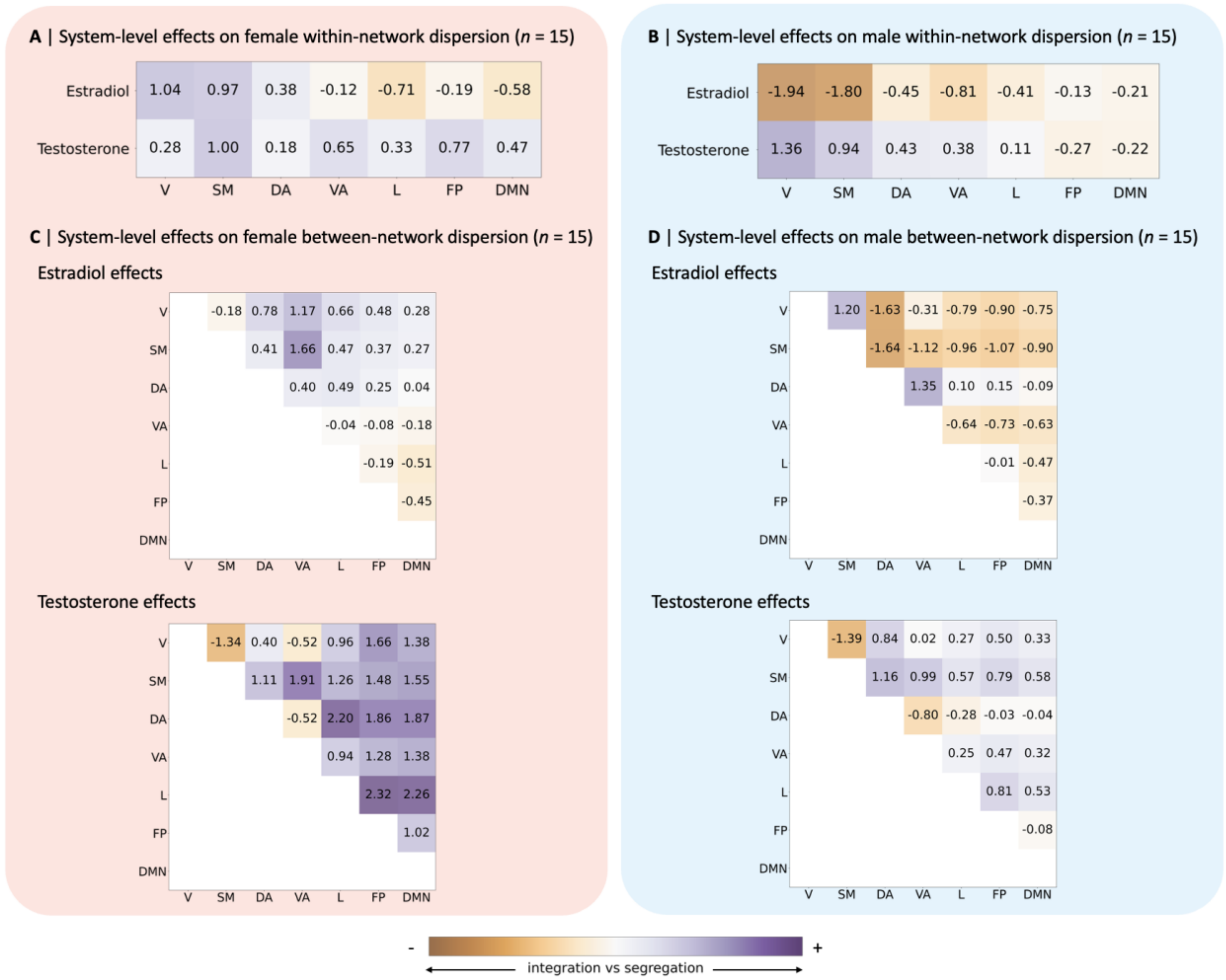

Supplementary Figure 9. System-level effects of estradiol and testosterone on within- and between-network dispersion for comparable female and male sample sizes (*n* = 15). For both sexes with equal sample sizes (*n* = 15), effects of common steroid hormones were tested in linear mixed effects models. Note that only the female results are novel (reduced sample) – the male results correspond to those reported in the main analyses. Heatmaps summarizing the *t*-values for system-level effects across functional networks of estradiol and testosterone for the female participant's within- (**A**) and between- (**C**) network dispersion, and the male participant's within- (**B**) and between- (**D**) network dispersion. *t*-values were obtained from linear mixed effects models including estradiol and testosterone as covariates in both the female and male models, tested separately per participant. None of the tested effects were statistically significant after correction for multiple comparisons, i.e., at Bonferroni-corrected thresholds of $p < 0.004$ (0.025/7) for the within-network effects and $p < 0.001$ (0.025/21) for the between network effects. Positive *t*-values represent higher segregation and negative *t*-values represent higher integration effects. V, visual; SM, somatomotor; DA, dorsal attention; VA, ventral attention; L, limbic; FP, frontoparietal; DMN, default-mode network.

Tables

	Estradiol			Progesterone		
**WN dispersion (network)**	***t***	***p***	***p*spin**	***t***	***p***	***p*spin**
Visual	0.327	0.744	-	0.609	0.543	-
Somatomotor	0.050	0.960	-	1.687	0.092	-
Dorsal attention	-0.784	0.433	-	2.053	0.040	-
Ventral attention	-1.150	0.250	-	0.943	0.346	-
Limbic	-0.887	0.375	-	1.161	0.246	-
Fronto parietal	-1.134	0.257	-	0.194	0.846	-
DMN	-1.662	0.097	-	1.038	0.299	-
**BN dispersion (pairwise networks)**	***t***	***p***	***p*spin**	***t***	***p***	***p*spin**
Visual - somatomotor	1.031	0.303	-	-1.743	0.081	-
Visual - dorsal attention	0.817	0.414	-	1.013	0.311	-
Visual - ventral attention	1.915	0.055	-	-1.027	0.305	-
Visual - limbic	-0.723	0.469	-	0.747	0.455	-
Visual - fronto parietal	-0.537	0.591	-	1.869	0.062	-
Visual - DMN	-0.516	0.606	-	1.385	0.166	-
Somatomotor - dorsal attention	-0.659	0.510	-	1.941	0.052	-
Somatomotor - ventral attention	0.874	0.382	-	2.189	0.029	-
Somatomotor - limbic	-0.940	0.347	-	1.442	0.149	-
Somatomotor - fronto parietal	-0.853	0.394	-	1.979	0.048	-
Somatomotor - DMN	-0.784	0.433	-	1.649	0.099	-
Dorsal attention - ventral attention	1.337	0.181	-	-1.398	0.162	-
Dorsal attention - limbic	-1.129	0.259	-	0.285	0.775	-
Dorsal attention - fronto parietal	-1.006	0.314	-	1.810	0.070	-
Dorsal attention - DMN	-1.037	0.300	-	1.275	0.202	-
Ventral attention - limbic	-1.490	0.136	-	1.044	0.296	-
Ventral attention - fronto parietal	-1.347	0.178	-	1.828	0.068	-
Ventral attention - DMN	-1.159	0.246	-	1.391	0.164	-
Limbic - fronto parietal	-0.411	0.681	-	4.371	0.000*	<.001
Limbic - DMN	-0.254	0.800	-	2.088	0.037	-
Fronto parietal - DMN	-0.010	0.992	-	-1.114	0.265	-

Supplementary Table 1. Steroid hormone effects on within-network (WN) and between-network (BN) dispersion in the female participant. Results are yielded by a linear mixed effects model including estradiol and progesterone as covariates. * indicates statistical significance after correcting for multiple comparisons, at Bonferroni-corrected thresholds of $p < 0.004$ (0.025/7) for the within-network effects and $p < 0.001$ (0.025/21) for the between network effects, followed by an additional correction for spatial autocorrelation via spin-permutation testing (1000 permutations), with $p_{spin} < 0.05$. DMN, default mode network.

	Testosterone			Cortisol		
**WN dispersion (network)**	***t***	***p***	***p*spin**	***t***	***p***	***p*spin**
Visual	-1.126	0.260	-	1.846	0.065	-
Somatomotor	-3.454	0.001*	0.001	2.637	0.008	-
Dorsal attention	0.376	0.707	-	0.815	0.415	-
Ventral attention	-0.211	0.833	-	1.462	0.144	-
Limbic	-0.518	0.604	-	0.573	0.567	-
Fronto parietal	-0.158	0.875	-	0.251	0.802	-
DMN	-0.410	0.682	-	0.836	0.403	-
**BN dispersion (pairwise networks)**	***t***	***p***	***p*spin**	***t***	***p***	***p*spin**
Visual - somatomotor	0.538	0.591	-	-2.517	0.012	-
Visual - dorsal attention	-3.550	0.000*	0.009	2.763	0.006	-
Visual - ventral attention	-2.459	0.014	-	-4.982	0.000*	<.001
Visual - limbic	-1.565	0.118	-	0.735	0.462	-
Visual - fronto parietal	-1.674	0.094	-	1.272	0.203	-
Visual - DMN	-1.557	0.120	-	1.024	0.306	-
Somatomotor - dorsal attention	-2.533	0.011	-	3.029	0.002	-
Somatomotor - ventral attention	-1.962	0.050	-	0.185	0.853	-
Somatomotor - limbic	-1.402	0.161	-	1.295	0.195	-
Somatomotor - fronto parietal	-1.460	0.144	-	1.776	0.076	-
Somatomotor - DMN	-1.410	0.159	-	1.451	0.147	-
Dorsal attention - ventral attention	1.744	0.081	-	-4.993	0.000*	0.003
Dorsal attention - limbic	-0.016	0.987	-	-1.142	0.254	-
Dorsal attention - fronto parietal	0.286	0.775	-	-0.268	0.789	-
Dorsal attention - DMN	-0.296	0.767	-	-0.016	0.987	-
Ventral attention - limbic	-0.674	0.500	-	1.489	0.136	-
Ventral attention - fronto parietal	-0.650	0.516	-	2.194	0.028	-
Ventral attention - DMN	-0.823	0.410	-	1.678	0.093	-
Limbic - fronto parietal	0.526	0.599	-	2.399	0.016	-
Limbic - DMN	-1.172	0.241	-	2.439	0.015	-
Fronto parietal - DMN	-1.084	0.278	-	0.336	0.737	-

Supplementary Table 2. Steroid hormone effects on within-network (WN) and between-network (BN) dispersion in the male participant. Results are yielded by a linear mixed effects model including testosterone and cortisol as covariates. * indicates statistical significance after correcting for multiple comparisons, at Bonferroni-corrected thresholds of $p < 0.004$ (0.025/7) for the within-network effects and $p < 0.001$ (0.025/21) for the between network effects, followed by an additional correction for spatial autocorrelation via spin-permutation testing (1000 permutations), with $p_{spin} < 0.05$. DMN, default mode network.

	PSS score (female)			PSS score (male)		
**WN dispersion (network)**	***t***	***p***	***p*spin**	***t***	***p***	***p*spin**
Visual	0.742	0.458	-	-1.962	0.050	-
Somatomotor	2.481	0.013	-	-1.257	0.209	-
Dorsal attention	1.446	0.148	-	-0.281	0.779	-
Ventral attention	1.167	0.243	-	-1.153	0.249	-
Limbic	1.573	0.116	-	-0.506	0.613	-
Fronto parietal	2.018	0.044	-	-0.660	0.509	-
DMN	0.891	0.373	-	-0.473	0.636	-
**BN dispersion (pairwise networks)**	***t***	***p***	***p*spin**	***t***	***p***	***p*spin**
Visual - somatomotor	-1.995	0.046	-	0.627	0.530	-
Visual - dorsal attention	-0.580	0.562	-	-1.480	0.139	-
Visual - ventral attention	-1.525	0.127	-	-1.331	0.183	-
Visual - limbic	1.005	0.315	-	-1.221	0.222	-
Visual - fronto parietal	1.266	0.206	-	-1.006	0.315	-
Visual - DMN	1.763	0.078	-	-0.852	0.394	-
Somatomotor - dorsal attention	1.590	0.112	-	-1.271	0.204	-
Somatomotor - ventral attention	1.917	0.055	-	-0.897	0.370	-
Somatomotor - limbic	1.574	0.115	-	-1.147	0.251	-
Somatomotor - fronto parietal	1.762	0.078	-	-0.964	0.335	-
Somatomotor - DMN	2.068	0.039	-	-0.852	0.394	-
Dorsal attention - ventral attention	-1.037	0.300	-	1.019	0.308	-
Dorsal attention - limbic	1.320	0.187	-	-0.472	0.637	-
Dorsal attention - fronto parietal	3.665	0.000*	<.001	-0.031	0.976	-
Dorsal attention - DMN	2.344	0.019	-	-0.193	0.847	-
Ventral attention - limbic	1.919	0.055	-	-0.939	0.348	-
Ventral attention - fronto parietal	2.506	0.012	-	-0.697	0.486	-
Ventral attention - DMN	1.911	0.056	-	-0.638	0.523	-
Limbic - fronto parietal	3.956	0.000*	.001	2.684	0.007	-
Limbic - DMN	3.215	0.001	-	1.083	0.279	-
Fronto parietal - DMN	2.618	0.009	-	-0.632	0.527	-

Supplementary Table 3. Perceived stress effects on within-network (WN) and between-network (BN) dispersion in the female and male participants. Results are yielded by independent linear mixed effects models for each participant, which only include perceived stress scale (PSS) score as a covariate. None of the tested effects were statistically significant after correction for multiple comparisons, i.e., at Bonferroni-corrected thresholds of $p < 0.004$ (0.025/7) for the within-network effects and $p < 0.001$ (0.025/21) for the between network effects. DMN, default mode network.

	Estradiol (female)			Estradiol (male)		
**WN dispersion (network)**	***t***	***p***	***p*spin**	***t***	***p***	***p*spin**
Visual	0.456	0.649	-	-1.941	0.052	-
Somatomotor	0.324	0.746	-	-1.798	0.072	-
Dorsal attention	-0.450	0.653	-	-0.453	0.651	-
Ventral attention	-0.870	0.384	-	-0.811	0.418	-
Limbic	-0.727	0.467	-	-0.410	0.682	-
Fronto parietal	-1.187	0.235	-	-0.128	0.898	-
DMN	-1.576	0.115	-	-0.211	0.833	-
**BN dispersion (pairwise networks)**	***t***	***p***	***p*spin**	***t***	***p***	***p*spin**
Visual - somatomotor	0.830	0.407	-	1.200	0.230	-
Visual - dorsal attention	0.924	0.356	-	-1.628	0.104	-
Visual - ventral attention	1.861	0.063	-	-0.312	0.755	-
Visual - limbic	-0.432	0.666	-	-0.787	0.431	-
Visual - fronto parietal	-0.236	0.813	-	-0.903	0.366	-
Visual - DMN	-0.260	0.795	-	-0.750	0.453	-
Somatomotor - dorsal attention	-0.356	0.722	-	-1.642	0.101	-
Somatomotor - ventral attention	1.201	0.230	-	-1.123	0.262	-
Somatomotor - limbic	-0.690	0.490	-	-0.957	0.339	-
Somatomotor - fronto parietal	-0.562	0.574	-	-1.066	0.286	-
Somatomotor - DMN	-0.563	0.573	-	-0.896	0.370	-
Dorsal attention - ventral attention	1.164	0.244	-	1.353	0.176	-
Dorsal attention - limbic	-1.328	0.184	-	0.098	0.922	-
Dorsal attention - fronto parietal	-0.838	0.402	-	0.152	0.879	-
Dorsal attention - DMN	-0.721	0.471	-	-0.089	0.929	-
Ventral attention - limbic	-1.288	0.198	-	-0.638	0.523	-
Ventral attention - fronto parietal	-1.063	0.288	-	-0.726	0.468	-
Ventral attention - DMN	-1.032	0.302	-	-0.631	0.528	-
Limbic - fronto parietal	0.306	0.760	-	-0.009	0.993	-
Limbic - DMN	0.085	0.932	-	-0.472	0.637	-
Fronto parietal - DMN	-0.190	0.849	-	-0.370	0.711	-

Supplementary Table 4. Estradiol effects on within-network (WN) and between-network (BN) dispersion in the female and male participants. Results are yielded by a linear mixed effects model including estradiol and testosterone as covariates. None of the tested effects were statistically significant after correction for multiple comparisons, i.e., at Bonferroni-corrected thresholds of $p < 0.004$ (0.025/7) for the within-network effects and $p < 0.001$ (0.025/21) for the between network effects. DMN, default mode network.

	Testosterone (female)			Testosterone (male)		
**WN dispersion (network)**	***t***	***p***	***p*spin**	***t***	***p***	***p*spin**
Visual	0.377	0.706	-	1.361	0.174	-
Somatomotor	2.033	0.042	-	0.938	0.348	-
Dorsal attention	1.724	0.085	-	0.434	0.664	-
Ventral attention	2.284	0.022	-	0.385	0.701	-
Limbic	1.626	0.104	-	0.107	0.915	-
Fronto parietal	1.687	0.092	-	-0.269	0.788	-
DMN	1.749	0.080	-	-0.219	0.827	-
**BN dispersion (pairwise networks)**	***t***	***p***	***p*spin**	***t***	***p***	***p*spin**
Visual - somatomotor	-2.844	0.004	-	-1.387	0.165	-
Visual - dorsal attention	-0.806	0.420	-	0.840	0.401	-
Visual - ventral attention	-2.648	0.008	-	0.019	0.985	-
Visual - limbic	0.994	0.320	-	0.265	0.791	-
Visual - fronto parietal	1.677	0.094	-	0.495	0.620	-
Visual - DMN	1.843	0.065	-	0.331	0.741	-
Somatomotor - dorsal attention	2.093	0.036	-	1.163	0.245	-
Somatomotor - ventral attention	1.602	0.109	-	0.988	0.323	-
Somatomotor - limbic	2.149	0.032	-	0.574	0.566	-
Somatomotor - fronto parietal	2.431	0.015	-	0.790	0.430	-
Somatomotor - DMN	2.526	0.012	-	0.581	0.561	-
Dorsal attention - ventral attention	-1.907	0.056	-	-0.804	0.421	-
Dorsal attention - limbic	1.838	0.066	-	-0.283	0.778	-
Dorsal attention - fronto parietal	2.685	0.007	-	-0.028	0.978	-
Dorsal attention - DMN	2.670	0.008	-	-0.039	0.969	-
Ventral attention - limbic	2.110	0.035	-	0.248	0.804	-
Ventral attention - fronto parietal	2.479	0.013	-	0.465	0.642	-
Ventral attention - DMN	2.595	0.009	-	0.317	0.752	-
Limbic - fronto parietal	2.478	0.013	-	0.811	0.418	-
Limbic - DMN	2.946	0.003	-	0.531	0.596	-
Fronto parietal - DMN	1.383	0.167	-	-0.080	0.936	-

Supplementary Table 5. Testosterone effects on within-network (WN) and between-network (BN) dispersion in the female and male participants. Results are yielded by a linear mixed effects model including estradiol and testosterone as covariates. None of the tested effects were statistically significant after correction for multiple comparisons, i.e., at Bonferroni-corrected thresholds of $p < 0.004$ (0.025/7) for the within-network effects and $p < 0.001$ (0.025/21) for the between network effects. DMN, default mode network.
